# Comparative performance of different scale‐down simulators of substrate gradients in *Penicillium chrysogenum* cultures: the need of a biological systems response analysis

**DOI:** 10.1111/1751-7915.13046

**Published:** 2018-01-15

**Authors:** Guan Wang, Junfei Zhao, Cees Haringa, Wenjun Tang, Jianye Xia, Ju Chu, Yingping Zhuang, Siliang Zhang, Amit T. Deshmukh, Walter van Gulik, Joseph J. Heijnen, Henk J. Noorman

**Affiliations:** ^1^ State key laboratory of Bioreactor Engineering East China University of Science and Technology (ECUST) Shanghai China; ^2^ Transport Phenomena Chemical Engineering Department Delft University of Technology Delft The Netherlands; ^3^ DSM Biotechnology Center Delft The Netherlands; ^4^ Cell Systems Engineering Department of Biotechnology Delft University of Technology Delft The Netherlands; ^5^ Bio Process Engineering Department of Biotechnology Delft University of Technology Delft The Netherlands

## Abstract

In a 54 m^3^ large‐scale penicillin fermentor, the cells experience substrate gradient cycles at the timescales of global mixing time about 20–40 s. Here, we used an intermittent feeding regime (IFR) and a two‐compartment reactor (TCR) to mimic these substrate gradients at laboratory‐scale continuous cultures. The IFR was applied to simulate substrate dynamics experienced by the cells at full scale at timescales of tens of seconds to minutes (30 s, 3 min and 6 min), while the TCR was designed to simulate substrate gradients at an applied mean residence time ($\tau_{c}$) of 6 min. A biological systems analysis of the response of an industrial high‐yielding *P. chrysogenum* strain has been performed in these continuous cultures. Compared to an undisturbed continuous feeding regime in a single reactor, the penicillin productivity (q_PenG_) was reduced in all scale‐down simulators. The dynamic metabolomics data indicated that in the IFRs, the cells accumulated high levels of the central metabolites during the feast phase to actively cope with external substrate deprivation during the famine phase. In contrast, in the TCR system, the storage pool (e.g. mannitol and arabitol) constituted a large contribution of carbon supply in the non‐feed compartment. Further, transcript analysis revealed that all scale‐down simulators gave different expression levels of the glucose/hexose transporter genes and the penicillin gene clusters. The results showed that q_PenG_ did not correlate well with exposure to the substrate regimes (excess, limitation and starvation), but there was a clear inverse relation between q_PenG_ and the intracellular glucose level.

## Introduction

In industrial practice, microbial fermentations are often operated as substrate‐limited, fed‐batch cultivations with high cell densities to obtain high volumetric productivities at low operational costs (Yamanè and Shimizu, 1984). The fed‐batch mode of operation is applied to keep the residual substrate concentration at a non‐repressing level, control biomass growth rate and product formation, and avoid oxygen limitation. To minimize broth dilution, a highly concentrated substrate solution is often fed via the top of the fermentor. Nonetheless, due to energy and construction constraints, there are relatively long transport distances in large‐scale fermentors (Wang *et al*., 2015). Together with high metabolic activity of the production microorganism, this feeding system gives rise to spatial substrate concentration gradients, that is zones with high substrate concentrations near the feeding point and zones with low substrate concentration further away (Larsson *et al*., 1996; Bylund *et al*., 1998; Lara *et al*., 2006). While travelling through the fermentor, the cells are therefore repeatedly subjected to oscillating substrate concentrations. This results in the specific substrate uptake rate (q_s_) and the growth rate (μ) with widely distributed values in the reactor. Simulation results of a 54 m^3^ industrial penicillin fermentation case show that three substrate regimes (excess, limitation and starvation) can be distinguished, where the regime average C_s_ values are $\bar{C_{s,\;\mathrm{starvation}}}$ ≈ 3.5 × 10^−3^ μM, $\bar{C_{s,\;\mathrm{limitation}}}$ ≈ 34.7 μM and $\bar{C_{s,\;\mathrm{excess}}}$ ≈ 294 μM, and the average substrate concentration in the reactor C_s, avg_ is about 34.4 μM (Haringa *et al*., 2016). For this large‐scale penicillin fermentor, the simulation result shows that about 55–60% of the culture (lower part of the vessel) experiences a famine regime and 5–10% of the culture (the upper part, close to the feeding inlet) experiences a feast regime, while the remaining 30–40% undergoes the limitation regime. The exposure of the cells to such substrate gradients is then triggers for cellular regulation and often results in performance loss (Larsson *et al*., 1996; Bylund *et al*., 1998; Enfors *et al*., 2001). However, in some cases, substrate gradients have contributed to a desired fungal morphology and resulted in a substantial increase of the productivities (Bhargava *et al*., 2003a,b,c). Hence, the uncertainty about the performance of the production organism in the full‐scale bioreactor imperils the competitiveness of biotechnological production processes (Lara *et al*., 2006; Takors, 2012).

**Figure 1:**
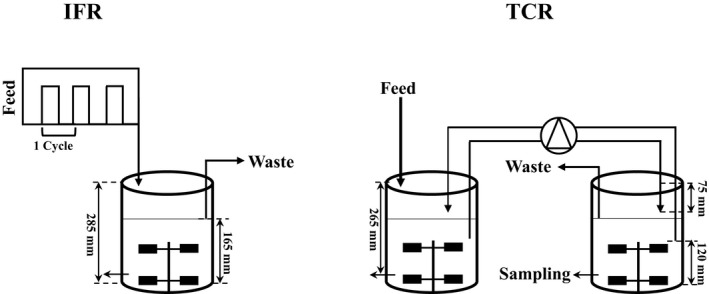
Scale‐down simulator configurations used. IFR, intermittent feeding regime; TCR, two‐compartment reactor.

To investigate the effects of large‐scale gradients, scale‐down (SD) and regime analysis are highly advocated to identify leads for improvement of industrial fermentation processes (Neubauer and Junne, 2010; Noorman, 2011; Wang *et al*., 2014; Delvigne and Noorman, 2017). In accordance, scale‐down studies have been used to unravel impaired cell performance, to elucidate regulatory mechanisms and to seek guidelines for strain and process engineering (Buchholz *et al*., 2014; Heins *et al*., 2015; Lemoine *et al*., 2015; Limberg *et al*., 2016, 2017; Loffler *et al*., 2016). Scale‐down studies on the effects of large‐scale substrate gradients can be performed in specific scale‐down simulators, for example, using an intermittent feeding regime (IFR) in a single reactor or a two‐compartment reactor (TCR) with broth recycle between the compartments whereby the substrate feed is supplied to one compartment (Fig. 1) (Wang *et al*., 2015). For example, in a scale‐down study with *P. chrysogenum* applying an IFR with a block‐wise feed/no feed regime (36 s on, 324 s off), de Jonge *et al*. (2011) observed a 50% decrease in penicillin productivity as well as a higher turnover rate of storage pools relative to continuously fed chemostat cultivation (de Jonge *et al*., 2011, 2014). However, as recently argued (Haringa *et al*., 2016), in these previous scale‐down studies, typically fluctuation timescales of 100–500 s have been applied (Vardar and Lilly, 1982; Neubauer and Junne, 2010; de Jonge *et al*., 2011; Heins *et al*., 2015; Lemoine *et al*., 2015), which were based on the 95% mixing time ($\tau_{95}$) at industrial scales (Limberg *et al*., 2016). However, a more realistic approach is to base the frequency of the substrate oscillations on the 4–5 times lower circulation time (Haringa *et al*., 2016), which implies that the cells in reality experience faster changes in substrate concentration at timescales of tens of seconds, which has been recently confirmed from computational fluid dynamics (CFD) simulations of the 54 m^3^ penicillin fermentation case (Haringa *et al*., 2016). Furthermore, it should be noted that the cells in an ideally mixed IFR are simultaneously exposed to the same conditions (Wang *et al*., 2014, 2015), which is not a reality in an actual fermentation process. At large scale, the conditions which the individual cells experience depend on the residence time distribution of the cells over the different zones of the fermentor (Sweere *et al*., 1988a,b; Neubauer and Junne, 2010). Fluctuations with a distributed duration can be realized using a TCR system (Lemoine *et al*., 2015; Limberg *et al*., 2017). In general, it appears that in most cases two distinct scale‐down simulators are used as follows: IFR and TCR. A particular variation of a TCR is that one compartment can be a stirred tank reactor (STR) while the other is a plug flow reactor (PFR). Surprisingly, very few studies have gathered information regarding differences in phenotype in IFR and TCR systems. To the best of our knowledge, there was only one scale‐down study by Sweere *et al*. (1988a,b) comparing the performance of *S. cerevisiae* in IFR and TCR, revealing that acetate and glycerol formation showed a distinct difference between both scale‐down simulator systems. However, a biological system analysis to explain such differences was not possible at that time.

In the present work, we compared the IFR and the TCR systems used to mimic substrate gradients as computed for the 54 m^3^ industrial‐scale fed‐batch penicillin fermentation process. All experiments were conducted in chemostat mode because a well‐defined, and controllable, set of physico‐chemical conditions can be maintained and reproduced (Hoskisson, 2005). As discussed elsewhere (Haringa *et al*., 2016), there are no indications that significant oxygen limitation has occurred in the 54 m^3^ industrial penicillin fermentation case and hence we only focused on the substrate gradients under the simulated industrial conditions. We performed a biological systems analysis (flux, intracellular metabolites and transcript) of the response of *P. chrysogenum* to the conditions in these SD simulators with glucose as the sole limiting substrate.

## Results and Discussion

To investigate the effect of dynamic substrate gradients, occurring in the 54 m^3^ industrial ‐scale penicillin fermentor (Haringa *et al*., 2016), on the metabolism and penicillin production of a high‐yielding *P. chrysogenum* strain, the strain was cultivated in the glucose‐limited chemostat set‐ups as IFR and TCR scale‐down simulators (Fig. 1). Also, reference chemostat cultivations with continuous feeding were carried out, representing continuous substrate‐limited conditions.

### General observations

This research strain in combination with the applied media and recipes did not cause wall growth and stickiness. Under the microscope, no significant morphological differences were observed among all these cultures. The DOT never dropped below 100% of air saturation in the reference chemostat cultivation and the IFRs, while in the TCR systems, the DOT remained at levels of 65% and 126% of air saturation in the feed compartment and non‐feed compartment, respectively.

### Penicillin production

**Figure 2:**
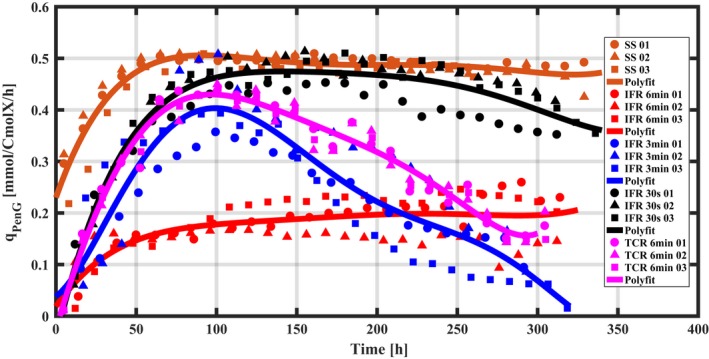
Time patterns of the biomass‐specific PenG production rate (q_PenG_) during glucose‐limited chemostat cultivations at the dilution rate of 0.05 h^−1^. Time 0 signifies the start‐up of the chemostat cultivations. Red, blue and black symbols represent the results of 6 min, 3 min and 30 s IFRs respectively. Brown symbol: reference conditions; pink symbol: the TCR systems (6 min). Each cultivation is represented by an individual symbol.

Time patterns of the biomass‐specific rates for penicillin production (q_PenG_) obtained for the reference condition, the IFRs and the TCR systems show that all triplicate continuous cultivations (chemostat, IFR and TCR) were highly reproducible (Fig. 2). Compared to the reference cultivations, there is a loss of the penicillin production capacity under dynamic feast/famine regimes, the extent of which largely depends on the cycle time. In the IFR system, a shorter cycle time leads to a penicillin productivity closer to that observed in the continuous feeding regime. In the TCR system, the q_PenG_ is also lowered compared to the chemostat but seems to be differently affected as compared to the IFR. For example, the q_PenG_ in the TCR ($\tau_{c}$ = 6 min) resembles that in the 3 min IFR (Fig. 2).

In the early phase of the reference cultivations, the specific penicillin production rate was observed to increase faster than in the IFR and TCR systems. A possible explanation for this phenomenon might be that the penicillin biosynthetic genes were less repressed because the C_s_ in the reference chemostat was much lower than the average values in the IFR and the TCR systems (See Extracellular Glucose Concentration).

### Stoichiometry

In all cultivations, carbon and degree of reduction balances were close to 100% (Table 1). In both scale‐down (SD) systems, the substrate consumption of *P. chrysogenum* can be described by the well‐known Herbert‐Pirt relation (Pirt, 1965; van Gulik *et al*., 2001). Because the average q_s_ values in the IFRs are about 10% higher (as a result of 10% less biomass), μ is nearly the same, and $q_{o_{2}}$ values are 10–15% higher, this implies that there are extra ATP losses. The origin of this loss is probably efficiency loss in metabolism, due to carry‐over of central metabolites from feast to famine (see below). Metabolic flux analysis, using a previously established stoichiometric model for growth and penicillin production in *P. chrysogenum* (van Gulik *et al*., 2000), shows that there is a 34% and 16% larger ATP gap in the IFRs of 6 min and 3 min, respectively, but in 30 s IFR, the ATP gap is nearly the same as in the chemostat cultures. Similarly, in *E. coli*, it has been found that the cellular energy demands for coping with fluctuating carbon or nitrogen source supply become higher under simulated large‐scale conditions, due to continuous on‐off switching of related genes and the increased cellular ATP costs of transcription and translation (Loffler *et al*., 2016, 2017; Simen *et al*., 2017).

**Table 1:** Reconciled biomass‐specific rates. These average rates were obtained from glucose‐limited cultures grown at a dilution rate of 0.05 h^−1^ at the time range of 100–200 h of chemostat cultivation

	Reference	IFR				TCR (6 min)	
		30 s	3 min	6 min		TCR1	TCR2
−q_s_	20.29±0.26	21.31±0.35	22.21±0.66	21.70±0.31	−q_s_	37.07±0.07	3.71±0.01
$-q_{O_{2}}$	55.13±0.45	56.10±2.00	65.98±3.20	60.05±1.60	$-q_{O_{2}}$	26.49±0.33	85.36±0.35
$-q_{{\mathrm{CO}}_{2}}$	59.00±0.46	59.82±2.00	69.34±3.20	63.38±1.60	$-q_{{\mathrm{CO}}_{2}}$	28.39±0.35	91.33±0.32
−q_PAA_	0.52±0.002	0.53±0.02	0.30±0.06	0.31±0.05	−q_PAA_	0.30±0.02	0.32±0.02
−q_PenG_	0.49±0.00	0.44±0.03	0.26±0.07	0.17±0.05	−q_PenG_	0.30±0.02	0.30±0.02
−q _Other excreted organic carbon_	14.37±0.78	15.17±0.61	12.07±0.71	16.58±0.45	−q _Stored carbon_	160.75±1.5	−160.75±1.5
μ_actual_	64.37±0.88	65.17±0.78	62.07±0.84	66.58±0.67	μ_actual_	28.39±0.06	91.33±0.07
C_X_	5.75±0.02	5.21±0.02	5.35±0.06	5.28±0.02	C_X_	5.71±0.07	5.74±0.04
C balance	98.32±0.76	95.57±1.61	97.29±2.39	99.63±1.30	C balance	96.27±0.68	101.12±0.17
γ balance	103.48±0.18	99.14±0.38	99.18±0.58	99.19±0.31	γ balance	98.81±0.17	99.93±0.11

Specific rates of biomass growth, other excreted organic carbon (in IFRs) and stored carbon (in TCR) are expressed in mCmol (Cmol h)^−1^. The biomass concentrations are expressed in g kg^−1^, and the other rates are all expressed in mmol (Cmol h)^−1^.

The TCR system shows the same biomass concentration as in the chemostat, but the average μ_actual_ is about 10% lower, indicating less biomass lysis. This suggests that the used broth recycle pump did not cause additional cell damage. Of interest is the $q_{o_{2}}$/q_s_ ratio, where this ratio in the IFRs and the reference chemostat is about 3 (molO_2_ molGlucose^−1^). However, the $q_{o_{2}}$/q_s_ ratios in the TCR1 and TCR2 are about 0.71 and 23.01 respectively. The first ratio shows that part of the consumed glucose in the TCR1 is not metabolized, while the second ratio indicates that there is more carbon (e.g. from the storage pool) in the TCR2 metabolized than carbon from glucose uptaken. This strongly suggests that there is carry‐over of metabolites between the TCR1 and TCR2.

### Extracellular glucose concentration

In the reference chemostat cultures, the extracellular C_s_ remains at the level of 2.6 μM over 250 h, and this agrees with the glucose uptake kinetics (Wang *et al*., 2017), giving a high glucose affinity of about 5 μM, in reasonable accordance with the 7.8 μM as reported by de Jonge *et al*. (2011). The extracellular C_s_ profiles in the IFRs are shown in Fig. S1. During the feed phase of each cycle, the C_s_ increased due to the addition of fresh medium, and then fast decreased linearly in time to virtually zero. However, the observed C_s_ and q_s_ dynamics in the 30 s IFR deviate from the model prediction (Figs S1 and S2), and there is clearly no famine regime. This system remains in the limitation regime. An explanation is that in this experiment there is an interference with the liquid circulation time of about 1–2 s (Wang *et al*., 2017), which is close to the feed switch‐on time interval (3 s) of the 30 s IFR. The maximum feast glucose concentrations in 30 s, 3 min and 6 min IFR experiments are 30, 167 and 333 μM (Fig. S1) respectively. Three metabolic regimes (excess, limitation and starvation) have been distinguished in the 54 m^3^ industrial‐scale penicillin fermentor; the maximum C_s_ value can reach 1500 μM around the feeding point, and the average regime C_s_ values are $\bar{C_{s,\;\mathrm{starvation}}}$ ≈ 3.5 × 10^−3^ μM, $\bar{C_{s,\;\mathrm{limitation}}}$ ≈ 34.7 μM and $\bar{C_{s,\;\mathrm{excess}}}$ ≈ 294 μM, respectively, as indicated in Fig. S1. Therefore, the starvation and excess glucose concentration levels present in the 54 m^3^ large‐scale fermentor are reasonably approached in the 3 min and 6 min IFRs used in this study, but the limitation regime is much smaller than at full‐scale. The 30 s IFR is fully in the limitation regime, but excess and starvation regimes are virtually absent (Fig. S1). In contrast, in the TCR system, when the $\tau_{c}$ = 6 min is applied, the glucose concentrations remain constant in each compartment (Fig. S3), and the glucose concentration difference between the two compartments is about 38 μM (Fig. S4). The total average $\bar{C_{s,\;\mathrm{avg}}}$ value in the 54 m^3^ large‐scale fermentor is about 34.4 μM (Haringa *et al*., 2016), which value is well in between the two compartments of the current TCR system (Fig. S4). The results show that the cells in both compartments will experience only limitation conditions if the $\tau_{c}$ = 6 min, but excess and starvation conditions are not achieved. It also should be noted that the broth passing through the broth exchange tubing will take about 9 s (2.6% × 360 s), and the glucose consumption times in the tubing are about 9 s (TCR1→TCR2) and 30 s (TCR2→TCR1). Therefore, the cells residing in the tubing will not experience serious starvation period.

Combining the above results, no clear relation between q_PenG_ and the C_s_ regimes (excess, limitation and starvation) was observed. However, Fig. S5 indicates that q_PenG_ in the IFRs seems to be inversely related to C_s, peak_ in the feast periods.

### Intracellular glucose concentration

The intracellular glucose levels (C_s, in_) show that the C_s, in_ values remain (over 250 h, Fig. S6) at the level of 0.8, 1.0 and 1.5 μmol gDW^−1^ in the 30 s, 3 min and 6 min IFRs, respectively, which are significantly higher than for the reference chemostat (about 0.125 μmol gDW^−1^). The measured dynamics of C_s, in_ during the IFR experiments (Fig. S7) indicates that C_s, in_ is rather independent of extracellular C_s_ or q_s_ while q_PenG_ seems to be also inversely related with the C_s, in_ level (Figs S8 and S9). Combing the inverse relation between q_PenG_ and C_s, peak_, it then shows that the C_s, in_ is linearly related to the C_s, peak_ in the IFRs (Fig. S10). More striking is that in the TCR system, the C_s, in_ shows utterly different dynamics, where the C_s, in_ in the TCR1 stays constant at about 2–3 μmol gDW^−1^, but the C_s, in_ in the TCR2 increases from 0.5–1 to 2–3 μmol gDW^−1^ after about 160 h (Fig. S11). The intra‐/extracellular glucose concentration ratio indicates that the glucose uptake system is not significantly changed in the TCR system after about 250 h of chemostat cultivation (Fig. S12).

### Glucose uptake

We assume that the specific glucose uptake rate (q_s_) during a complete cycle is a hyperbolic function of C_s_, according to q_s_ = $q_{s}^{\max}\left(\frac{C_{s}}{K_{s}+C_{s}}\right)$. With the estimated parameters $q_{s}^{\max}$ = 0.0417 mol CmolX^−1 ^h^−1^ and K_s_ = 7.8 μM (Tang *et al*., 2017), the q_s_ profiles in the IFRs of 3 min and 6 min can be reproduced very well (Fig. S2), but not in the 30 s IFR. In the TCR system, the residual glucose concentration C_s_ level (57 μM) is threefold higher in TCR1 (feed compartment) than in TCR2 (non‐feed compartment) (19 μM) (Fig. S4). From the steady state substrate balance for the TCR system, it is calculated that the specific glucose uptake rate of the cells in the feed compartment, q_s, TCR1_ is 37 mmol CmolX^−1^ h^−1^ while the specific glucose uptake rate in the non‐feed compartment q_s, TCR2_ is 10 times lower (3.7 mmol CmolX^−1^ h^−1^) (Table 1). Consistent with the lower q_s_ values in the TCR2, the intracellular levels of the metabolites in the glycolysis and the TCA cycle are lower in the TCR2 than in the TCR1 (Table S1). However, it should be noted that when the hyperbolic glucose uptake kinetic model (obtained from the chemostat and the IFR experiments) is used for calculation of q_s_ values in the TCR system, the values in the q_s, TCR1_ and q_s, TCR2_ are 36.7 and 29.6 (mmol CmolX^−1^ h^−1^) respectively. The value in the non‐feed compartment is very different from that calculated using the substrate balance. This suggests that the substrate uptake kinetics in the TCR system have changed to a lower affinity (K_m_ value of about 25 μM). This change of affinity is also supported by the reduced mRNA levels of most of the glucose/hexose transporter genes (Transcript Analysis).

### Carry‐over of metabolites in IFR

**Figure 3:**
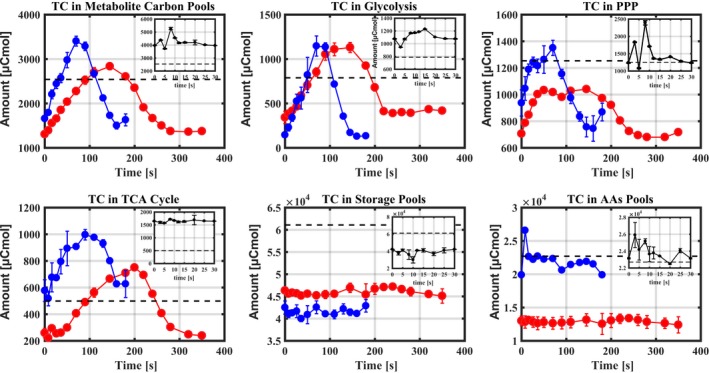
Categorized carbon pool dynamics in the central metabolism in the IFRs.TC: total carbon in the whole vessel; Red, blue and black dots represent the results of 6 min, 3 min and 30 s IFRs respectively. The dashed line represents the average level of the continuous feeding regime. TC in ‘metabolite carbon pool’ contains the total carbon in glycolysis, PPP and TCA cycle.

As expected, the dynamic feast/famine regimes with the feed cycles of 3 min and 6 min lead to most central metabolites following residual glucose/G6P dynamics, but the pools of the hexose‐phosphates show delayed dynamics and start to decrease when the C_s_ is still in excess (Fig. S13). Mass action ratios (MARs) for phosphoglucose isomerase (PGI), enolase, phosphomannose‐isomerase (PMI) and fumarase are all changed within a feast–famine cycle (Fig. S14). In the IFR systems, these MARs increased (especially PGI) above their equilibrium values in the famine phase, showing that flux reversed because central metabolites are consumed. This can only occur when these metabolites accumulate in the feast phase. The organic carbon present in the measured metabolites inside the cells can be categorized as carbon pools of glycolysis, TCA cycle, PPP, storage and amino acids (Fig. 3). Surprisingly, the maximum amount of carbon accumulated as central metabolites during the feast phase (Fig. 3) is about 20% higher in the 3 min IFR than in the 6 min IFR. During the 3 min IFR, from 0 to 70 s (feast phase), the total metabolite carbon in the vessel (in μCmol) in glycolysis, TCA cycle and PPP increased by about 1750 μCmol, while during the 6 min IFR, from 0 to 145 s (feast phase), the total carbon (in μCmol) in these pools increased by about 1530 μCmol (Fig. 3). To maintain a balanced system, this implies in the famine phase that in the 3 min and 6 min IFRs there is consumption of intracellular pools of 1.75 and 1.53 mmolC respectively. The glucose concentration levels (Fig. S1) in the 3 min and 6 min IFRs show that at the start of the feast phase there is about 0.167 and 0.333 mM glucose present, that is, about 3 and 6 mmolC in the whole vessel respectively. Hence, 58% (1.75/3) and 26% (1.53/6) of the substrate carbon seem to accumulate as metabolites in the feast phase of the 3 min and 6 min IFRs, before entering into the famine phase. In contrast, no significant carbon dynamics was observed in pools of storage carbohydrates and amino acids within a complete feeding cycle from feast to famine phase (Fig. 3). In addition, during the complete cycles in the IFRs of 3 min and 6 min, the summed non‐glucose extracellular carbons vary between 20 and 50 μCmol in the whole vessel (data not shown), respectively, which are far lower than the above discussed feast/famine carbon exchange of about 1600 μCmol. Therefore, this part of carbon was not included in the carbon balance analysis. Although the metabolites in the 30 s IFR do not show strong dynamics (Fig. S13) because of the very short time window and the liquid circulation time (about 1–2 s) of the applied bioreactor, being close to the feed period of 3 s, from 0 to 8 s, the total amount of carbon (in μCmol) accumulated in the form of metabolites from glycolysis, TCA cycle and PPP was 5270 μCmol (Fig. 3), which is about 1.5 times and 1.9 times higher than the peak values in the 3 min and 6 min IFRs respectively.

A key question now is as follows: Can the carbon that is accumulated in the feast phase sustain the carbon consumption in the famine phase? It should be first noted that the residual glucose concentration in the 30 s IFR was much less dynamic compared to the 3 min and 6 min IFR's and its value is between 14 and 30 μM during the complete feeding cycle (Fig. S1). As a consequence, the cells exposed to this feeding profile are always in the limitation regime and do not undergo serious feast nor famine phases. For the IFRs of 3 min and 6 min, a full carbon balance will shed light on the available carbon to sustain metabolic activities in the famine phase. Reconstruction of the CO_2_ production rate from off‐gas information is complicated by probe dynamics, head space buffering and lag time in the tubing system before the off‐gas enters the mass spectrometry. Here, we used a recently published 9‐pool model (Tang *et al*., 2017) to obtain off‐gas CO_2_ dynamics (Fig. S15) and then the total carbon emission as CO_2_ (in μCmol) can be estimated. We observed that during the famine phase in the 3 min and 6 min IFRs, the model‐predicted carbon emission as CO_2_ was about 663 and 1098 μCmol respectively. The accumulated amounts of carbon in the 3 min and 6 min IFRs were about 1600 μCmol, which is far higher than the total carbon emitted as CO_2_, therefore, the metabolite carbon accumulated in the feast phase is enough to sustain carbon consumption in the famine phase under these feeding regimes. This makes sense as the cells need to coordinate their metabolism to deal with changing environments, failure to do so will result in growth arrest and/or imbalanced state (van Heerden *et al*., 2014).

### Carry‐over of metabolites in TCR

In contrast to the IFRs where feast/famine carbon carry‐over is mainly as intracellular central carbon metabolites, in the TCR system, besides carbon in the metabolite carbon pool, there seems to be major contribution of storage pools, such as mannitol and arabitol, as the carbon carry‐over (Table S2). Table S2 shows that compared to the reference chemostat, higher intracellular trehalose and lower intracellular sugar alcohols are observed in the IFRs, while, lower intracellular trehalose and higher intracellular sugar alcohols are observed in the TCR system.

**Figure 4:**
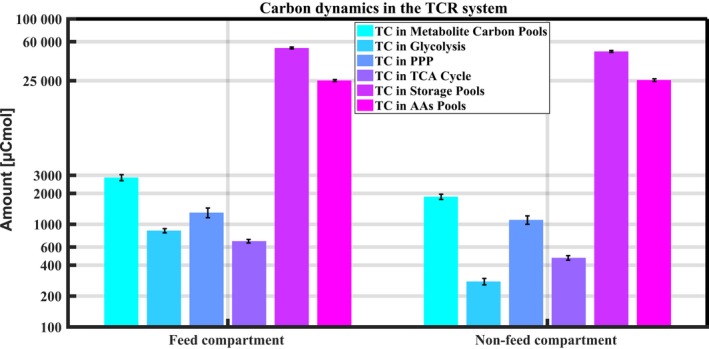
Categorized carbon pool dynamics in the central metabolism in the TCR system.

In the TCR system, the total carbon in the metabolite carbon pool is about 998 μCmol higher in the TCR1 (2850 μCmol) than in the TCR2 (1852 μCmol) (Fig. 4). The total carbon in storage pools is about 3895 μCmol higher in the TCR1 than in the TCR2. The carbon consumed in TCR2 from the metabolite pool and the storage pool amount to 20 and 78 mmolC h^−1^ respectively. The glucose consumed in the TCR2 is about 2.45 mmol h^−1^, which represents about 9% of the total feed (27 mmol h^−1^) in the TCR1. Combining for TCR2 the glucose consumed carbon, differences in the excreted metabolites (Fig. S16), the intracellular carbon pools of metabolites, storage carbohydrates and amino acids, shows that the total carbon disappearing in the TCR2 is about 100 mmolC h^−1^, representing about 60% of the total feed (162 mmolC h^−1^). This shows that the massive carry‐over of carbon from feed (TCR1) to non‐feed compartment (TCR2) in the TCR simulator is mostly as storage metabolites, which is in contrast to the IFRs, where carry‐over is mostly as central carbon metabolites. Surprisingly, this stored carbon pool is accumulated in the TCR1 and thereby consumed in the TCR2 at a high value of about 160 mmolC CmolX^−1^ h^−1^ (Table 1). However, it is reasonable because the stored carbohydrate pool is abundant in mannitol and arabitol (Table S2); it would be about 0.17 g g^−1^ DCW h^−1^ in terms of consumed glucose carbon, which could also be possible.

In the TCR2, the PEP level and the pyruvate level are not much reduced compared to other glycolytic intermediates such as G6P, F6P, suggesting there might be a buffering capacity of the closely related amino acid, alanine (Suarez‐Mendez *et al*., 2014), which is indeed observed to be 14% lower in the non‐feed compartment (Table S3). Besides this, no significant differences are observed regarding the intracellular levels of amino acids between the continuous feeding regime, the IFRs and the TCR system (Table S3).

### Transcript analysis

**Figure 5:**
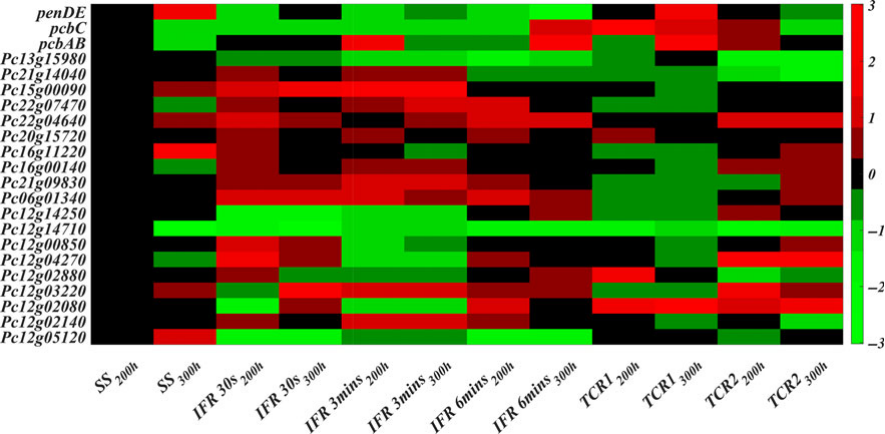
Heat map of the transcript level of three penicillin biosynthetic genes (*pcbAB*,* pcbC* and *penDE*) and 19 putative glucose/hexose transporter genes (the rest) over the cultures age at the reference chemostat conditions, the IFRs and the TCR systems. All data are normalized to the data at 200 h of the reference chemostat.

In previous studies, it was found that the transcription of the penicillin gene cluster is repressed by extracellular glucose (Revilla *et al*., 1986; Feng *et al*., 1994; Brakhage, 1998; Gutiérrez *et al*., 1999; Litzka *et al*., 1999; Martín *et al*., 1999). Further, Wang *et al*. (2017) observed that a very low C_s_ may induce the expression of the high‐affinity glucose transporter genes, and the related glucose sensing can trigger metabolic rearrangement, for example futile cycling, which leads to an extra ATP consumption and eventually results in the decrease of penicillin production. Therefore, in this study, the transcript levels of the glucose/hexose transporter genes have been determined. The transcript levels of these genes were measured at 200 and 300 h of chemostat cultures under the reference conditions, the IFRs and the TCRs. All transcript data were normalized to the transcript levels at 200 h in the reference chemostat. For cultivations in the IFRs, the transcript levels of the most of these glucose/hexose transporter genes are increased from 200 to 300 h (Fig. 5). For the TCR system, from 200 to 300 h, the transcript levels of most of these glucose/hexose transporter genes in the TCR1 are drastically decreased, while most of these transcript levels do not show a significant change in the TCR2 (Fig. 5). The transcript results in the TCR system are also consistent with the higher estimated glucose affinity. This difference can be explained by the different C_s_ profiles (Fig. S1). Because in the 3 min and 6 min IFR cycles, the cells experience periods where C_s_ is virtually zero, this may trigger the glucose‐related sensing and a continued expression of the glucose/hexose transporter genes. In contrast, while the cells experience a jump in C_s_ in the TCR system, the lower C_s_ in the TCR2 (19 μM) is still sufficiently high to avoid such triggering towards high expression, and as a result the expression of transporter genes declines. Further, the decrease of the penicillin production must be linked to the expression levels of the penicillin gene cluster (Douma *et al*., 2010; Deshmukh *et al*., 2015). Striking is that the expression of the penicillin gene cluster does not show a uniform trend and the decrease of the penicillin production must be ascribed to different expression levels of the penicillin pathway genes, that is, the *pcbAB*, the *pcbC* and the *penDE* genes. For the 6 min IFR, the transcript level of the *penDE* gene encoding the isopenicillin *N* acyltransferase (IAT) was 70 times lower at 300 h than at 200 h. While, for the 3 min IFR, the transcript level of the *pcbAB* gene encoding the L‐α‐(δ‐aminoadipyl)‐L‐α‐cysteinyl‐D‐α‐valine synthase (ACVS) was about six times lower in the 300 h than in the 200 h. In the 30 s IFR, from 200 to 300 h, we observed that the transcript level of the *pcbAB* gene was slightly increased, while at the same time the transcript levels of the *pcbC* gene and the *penDE* gene were slightly decreased. However, in the TCR system ($\tau_{c}$ = 6 min), the *pcbC* gene encoding the isopenicillin *N* synthase (IPNS) was about two times lower expressed at 300 than at 200 h. Therefore, different IFRs and the TCR system may induce largely differential expression levels of the penicillin gene clusters, and thereby differences in penicillin productions.

In summary, the penicillin productivity was reduced in all scale‐down simulators, that is, the IFR and the TCR systems, relative to an undisturbed, continuous chemostat condition. However, there is no clear relation to the exposure to substrate regimes (excess, limitation and starvation). There is a clear inverse relation between q_PenG_ and C_s, in_. *P. chrysogenum* showed different metabolic strategies to cope with different (dynamic) substrate gradient cycles. In the feast–famine regimes of the IFRs, the cells accumulated the surplus carbon in the form of the central metabolites, rather than in carbohydrate storage pools. In the IFRs, shorter feeding cycles led to, as expected, penicillin productivities closer to that observed in the continuously fed reference chemostat. The penicillin productivity was found to be less affected in the TCR system with two different regions of substrate levels as compared with the IFRs. Further, the metabolomics data show that the storage pools (e.g. mannitol and arabitol) constitute a big contribution in the carbon supply in the non‐feed compartment. Different IFRs and the TCR system induce differential expression levels of the glucose transporter genes and the penicillin gene clusters, which result in different penicillin productions. This study shows that in order to secure that scale‐down simulators deliver results representative for large‐scale conditions, design of these simulators should be guided by a detailed systems analysis of the large‐scale and scale‐down simulators.

## Experimental procedures

### Strain and medium

A high‐yielding strain of *P. chrysogenum*, DS17690, was used for all experiments, and its characteristics have been reported elsewhere (van Gulik *et al*., 2000; Nasution *et al*., 2006; Harris *et al*., 2009). This strain was kindly donated by DSM Sinochem Pharmaceuticals (Delft, The Netherlands) as spores on rice grains. Preparation and sterilization of batch and chemostat medium have been described previously (van Gulik *et al*., 2000; de Jonge *et al*., 2011).

### Chemostat cultivation conditions

All cultivations used identical 3 l working volume fermentors, where the TCR consisted of two of these vessels connected by broth recycling through a peristaltic pump (Longer Pumps, YT600‐1J, 0.6–11 l min^−1^). All chemostat cultivations were controlled at a dilution rate of 0.05 h^−1^ (150 ml h^−1^ feed in IFR and the reference chemostat conditions and 300 ml h^−1^ in the feed compartment of the TCR, with the same glucose concentration of 15 g l^−1^ in the feeds), a gas flow rate of 2 l min^−1^ in each vessel, and 0.5 bar overpressure. Effluent was removed by a peristaltic pump through an overflow pipe positioned at the gas/liquid interface of the bioreactors. The temperature was kept at 25 °C, and the broth was stirred (400 RPM) by two‐six‐bladed Rushton turbine stirrers (D = 72 mm, mutual impeller clearance, 72 mm). The DOT was measured *in situ* with a sterilizable oxygen probe (Mettler‐Toledo, Urdorf, Switzerland) (calibration at 150% (12.8 mg O_2_ l^−1^) at 0.5 bar overpressure), and the pH of the culture system was maintained at 6.5 by automatically adding 4 M NaOH and using a sterilizable pH probe (Mettler‐Toledo) mounted in the bioreactor. The off‐gas oxygen and carbon dioxide levels were real‐time monitored by off‐gas mass spectrometry (MAX300‐LG; Extrel, Pittsburgh, PA, USA). For the TCR system, the compositions of the inlet and outlet gasses of the two compartments were separately monitored. A scheme of the location of entrance and removal of circulating broth, sampling port, feed inflow and broth outflow as well as the broth recycle pump is shown in Fig. 1.

Reference chemostat cultivations with constant substrate feeding were performed in triplicate. Scale‐down experiments were carried out in: (i) the IFR system at three different cycle times, each was performed in triplicate. In this system, on/off feeding was applied with different cycle times, where the feed period was always 10% of the total on/off cycle. During the feeding interval, the pump speed was set 10 times higher than that under reference conditions to keep the average glucose feeding rate of the intermittently fed cultures the same as that of the control chemostats. The feed pump was precisely controlled by a timer, switching it on every first 3, 18 and 36 s of the cycle for cycle times of 30 s, 3 min and 6 min, respectively; (ii) the TCR system, in which also all experiments were performed in triplicate. Both vessels are connected by 2 m tubing (Φ = 1 cm) wherein the broth volume is 157 ml, about 2.6% of the total 6 l broth. In this system, the substrate concentrations in each ideally mixed compartment are determined by the broth recycle rate. Knowing that the full‐scale simulated mean circulation time of the 54 m^3^ penicillin reactor is about 19 s (Haringa *et al*., 2016), it was calculated that substrate limitation and starvation regions as experienced by the cells in the large‐scale penicillin fermentation case can only be obtained with at least an industrial biomass concentration (approx. 50–60 g kg^−1^ dry matter). However, mass transfer will then become a problem in laboratory‐scale reactors, and high biomass concentrations will impede fast sampling and quenching procedures. Therefore, in this study, we mainly aimed to investigate the effect of a volume‐based C_s_ distribution on cell metabolism and penicillin production. According to the simulation results of the 54 m^3^ industrial‐scale penicillin broth, the volumetric distribution of non‐starvation (excess plus limitation) and starvation is roughly the same (Haringa *et al*., 2016). Therefore, we used an equal volume distribution for the TCR in this study. The medium feed (300 ml h^−1^) was supplied to one compartment while the effluent was removed from the other compartment. The broth was circulated (60 l h^−1^, the flow was premeasured using the balance) in between the two compartments, and therewith the total mean residence time was set at 6, with 3 min in each compartment.

### Rapid sampling

Rapid sampling was carried out for acquisition of snapshots of both intracellular and extracellular metabolites and as well for analysis of mRNA levels. The detailed procedures can be found in supporting information.

### Calculation methods

The biomass‐specific rates (or q‐rates) were calculated from the corresponding compound balances. The $q_{O_{2}}$ and $q_{CO_{2}}$ in the IFRs were the average values in the chemostat cultivations of 100–200 h. The calculation of q‐rates in the TCR system can be found in Supporting information. The thus obtained q‐rates were reconciled under the constraint that the elemental conservation relations were satisfied, using the approach of (Verheijen, 2010).

### Analytical procedures

Metabolites of the glycolytic pathway, the TCA cycle and the PP pathway were quantified by GC‐MS using the isotope dilution mass spectrometry (IDMS) method, as described previously (Cipollina *et al*., 2009; de Jonge *et al*., 2011). Concentrations of phenylacetic acid (PAA) and penicillin G (PenG) in the filtrate were determined by high‐pressure liquid chromatography (HPLC) as described previously (Harris *et al*., 2007). Organic carbon in the total broth (TOC_broth_) and filtrate (TOC_supernatant_) was analysed by a TOC analyser (TOC‐5050A, Shimadzu) according to the manufacturers’ instructions.

### 9‐pool metabolic structured kinetic model

A 9‐pool metabolic structured kinetic model was used for predicting the dynamic off‐gas carbon dioxide levels for the IFR experiments (Tang *et al*., 2017). The modelling procedure can be found in the Supporting information.

## Conflict of interest

None declared.

## Supporting information


**Fig. S1.** Residual glucose concentration (C_s_) profiles in the IFRs.
**Fig. S2.** Specific glucose uptake rate (q_s_).
**Fig. S3.** Extracellular C_s_ values as a function of time in the TCR system.
**Fig. S4.** Average residual glucose concentrations in the TCR system are based on 10 independent data points.
**Fig. S6.** Intracellular glucose levels as function of the culture age in the IFRs.
**Fig. S7.** Intracellular glucose levels over a complete feed cycle in the IFRs.
**Fig. S8.** q_penG_ against C_s, in_ in the IFRs.
**Fig. S9.** q_penG_ against C_s, in_ in the TCR2.
**Fig. S10** C_s, in_ against C_s, peak_.
**Fig. S11.** Intracellular glucose levels as function of the culture age in the TCR.
**Fig. S12.** The intra/extra‐cellular glucose concentration ratio in the TCR system, where 2.5 ml gDW^−1^ is assumed for the conversion.
**Fig. S13.** Concentration measurements of metabolites (in μmol^−1^gDW^−1^) in the glycolysis, PPP, TCA cycle and storage pools.
**Fig. S14.** Mass action ratios for phosphoglucose isomerase (PGI), enolase, mannose‐6‐phosphate isomerase (PMI) and fumarase.
**Fig. S15.** The biomass specific carbon emission rates within a complete feeding cycle predicted by the 9‐pool model (Tang *et al*., 2017).
**Fig. S16.** Extracellular metabolites in the IFRs and the TCR system.
**Fig. S17.** Overview of the 9‐pool model for *P. chrysogenum*.
**Table S1.** Average intracellular amounts (in μmol gDW^−1^) of glycolytic, PPP, TCA cycle intermediates measured based on three individual chemostat cultivations within the time range of 100 h to 200 h in the continuous feeding and TCR system.
**Table S2.** Average Storage carbohydrates measured (in μmol gDW^−1^) based on three individual chemostat cultivations within the time range of 100 h to 200 h in the continuous feeding, the IFRs and the TCR system.
**Table S3.** Average intracellular levels of amino acids measured (in μmol gDW^−1^) based on three individual chemostat cultivations within the time range of 100 h to 200 h in the IFRs and TCR system.
**Table S4.** Complete stoichiometric matrix of the metabolically structured kinetic model (Tang *et al*., 2017).
**Table S5.** Kinetics used in the metabolically structured kinetic model (Tang *et al*., 2017).
**Table S6.** Optimized parameters used in the kinetics of the metabolically structured model (Tang *et al*., 2017).
