# Supplementary material for: Comparative performance of different scale‐down simulators of substrate gradients in Penicillium chrysogenum cultures: the need of a biological systems response analysis
Source: Microb Biotechnol. 2018 Jan 15;11(3):486–97. doi: 10.1111/1751-7915.13046 (PMC5902331; doi:10.1111/1751-7915.13046)
Supplement: Supplementary file 1 — Fig. S1. Residual glucose concentration (Cs) profiles in the IFRs. Fig. S2. Specific glucose uptake rate (qs). Fig. S3. Extracellular Cs values as a function of time in the TCR system. Fig. S4. Average residual glucose concentrations in the TCR system are based on 10 independent data points. Fig. S6. Intracellular glucose levels as function of the culture age in the IFRs. Fig. S7. Intracellular glucose levels over a complete feed cycle in the IFRs. Fig. S8. qpenG against Cs, in in the IFRs. Fig. S9. qpenG against Cs, in in the TCR2. Fig. S10 Cs, in against Cs, peak. Fig. S11. Intracellular glucose levels as function of the culture age in the TCR. Fig. S12. The intra/extra‐cellular glucose concentration ratio in the TCR system, where 2.5 ml gDW−1 is assumed for the conversion. Fig. S13. Concentration measurements of metabolites (in μmol−1gDW−1) in the glycolysis, PPP, TCA cycle and storage pools. Fig. S14. Mass action ratios for phosphoglucose isomerase (PGI), enolase, mannose‐6‐phosphate isomerase (PMI) and fumarase. Fig. S15. The biomass specific carbon emission rates within a complete feeding cycle predicted by the 9‐pool model (Tang et al., 2017). Fig. S16. Extracellular metabolites in the IFRs and the TCR system. Fig. S17. Overview of the 9‐pool model for P. chrysogenum. Table S1. Average intracellular amounts (in μmol gDW−1) of glycolytic, PPP, TCA cycle intermediates measured based on three individual chemostat cultivations within the time range of 100 h to 200 h in the continuous feeding and TCR system. Table S2. Average Storage carbohydrates measured (in μmol gDW−1) based on three individual chemostat cultivations within the time range of 100 h to 200 h in the continuous feeding, the IFRs and the TCR system. Table S3. Average intracellular levels of amino acids measured (in μmol gDW−1) based on three individual chemostat cultivations within the time range of 100 h to 200 h in the IFRs and TCR system. Table S4. Complete stoichiometric matrix of the metabolica [file MBT2-11-486-s001.docx]

# SUPPLEMENTARY MATERIALS:

***Comparative Performance of Different Scale-down Simulators of Substrate Gradients in Penicillium chrysogenum Cultures: The Need of a Biological Systems Response Analysis***

# Author names and affiliations:

Guan Wang^1^, Junfei Zhao^1^, Cees Haringa^2^, Wenjun Tang^1^, Jianye Xia^1^, Ju Chu^1*^, Yingping Zhuang^1^, Siliang Zhang^1^, Amit T. Deshmukh^3^, Walter van Gulik^4^, Joseph J. Heijnen^4^, Henk J. Noorman^3,5^

1. State key laboratory of Bioreactor Engineering, East China University of Science and Technology (ECUST), Shanghai, People’s Republic of China.
2. Transport Phenomena, Chemical Engineering Department, Delft University of Technology, Delft, The Netherlands.
3. DSM Biotechnology Center, Delft, The Netherlands.
4. Cell Systems Engineering, Department of Biotechnology, Delft University of Technology, Delft, The Netherlands.
5. Bio Process Engineering, Department of Biotechnology, Delft University of Technology, Delft, The Netherlands.

*Corresponding author. E-mail [juchu@ecust.edu.cn](mailto:juchu@ecust.edu.cn)

**Sampling Procedures**

**Sampling for determination of cell dry weight and total organic carbon**

An amount of 15 mL broth was withdrawn and split in triplicate for measurement of cell dry weight (CDW), using glass fiber filters (type A/E; Pall Corporation, East Hills, NY; 47 mm in diameter, 1-μm pore size), pre-dried overnight at 70 °C. For a CDW sample, 5 mL broth was filtered and the cell cake was washed twice with 10 mL demineralized water, and dried at 70 °C for 24 h. The biomass-containing filters were cooled to room temperature in a desiccator before weighing. Another 10 mL broth and 10 mL supernatant were stored at -20 °C for analysis of the total organic carbon (TOC) concentration in the broth and in the supernatant with a TOC analyzer (TOC-5050A, Shimadzu).

**Rapid sampling and quenching for analysis of extracellular glucose and other excreted metabolites**

The cold steel-bead method combined with liquid nitrogen was efficiently used for fast filtration and quenching of extracellular enzyme activities (Mashego, et al., 2003, de Jonge, et al., 2011). About 32 grams of stainless steel beads (4 mm diameter) was stored in a syringe and precooled to -20 °C. Approximately 1 mL broth was transferred from the bioreactor into the syringe for cooling of the sample within a fraction of a second to close to 0 °C and then was rapidly filtered through a Millex HV 0.45 μm filter (Millipore, Billerica, MA). For absolute determination of extracellular metabolites, 2 μL of 100 mM N-Ethylmaleimide (NEM) for thiol group protection (Seifar, et al., 2012) and 20 μL of U-^13^C-labeled cell extract (Mashego, et al., 2004, Wu, et al., 2005) as an internal standard (IS) were added before the filtrate sample was quickly frozen in liquid nitrogen. Subsequently the sample was stored at -80 °C until analysis.

**Rapid sampling, quenching and subsequent extraction for analysis of intracellular metabolites**

To obtain snapshots of intracellular metabolites, samples were obtained within half a second by rapidly withdrawing about 1 mL of broth from the bioreactor, using a custom-made rapid sampling device, into a tube containing 8 mL 40% (v/v) methanol/water mixture at -27.5 °C for instantaneous quenching of the cell metabolism (de Jonge, et al., 2012). The exact sample weights were determined by weighing all tubes before and after sampling. Fast filtration and a modified cold washing method were used for rapid and effective removal of all compounds present outside the cells (Douma, et al., 2010). The boiling ethanol method was adopted for rapid and reliable extraction of intracellular metabolites (Gonzalez, et al., 1997). Prior to being exposed to the boiling solution, 24 μL of NEM and 120 μL of IS were added to the sample. According to the method, 30 mL of 75% (v/v) ethanol/water mixture (pre-heated at 75 °C) was then transferred to the cell pellet, followed by resuspension of the pellet and the sample tube was incubated in a 95 °C water bath for 3 min. Afterwards, the cell extract/ethanol solution was cooled on ice and subsequently concentrated in a Rapid-Vap (Labconco, Kansas City, MO) under controlled vacuum and room temperature to reach a final volume of approximately 300 μL. Before mass spectrometry-based analysis, the concentrated cell extracts were quantitated to 600 mg by adding Milli-Q water and filtered with a Millex HV 0.22 μm filter (Millipore, Billerica, MA) to remove cell debris. The filtrate was then stored at -80 °C pending further analysis.

**Sampling for qPCR**

Samples were rapidly taken, filtered and the biomass was immediately quenched in liquid nitrogen. Upon analysis, RNA was isolated and double stranded cDNA was synthesized as described elsewhere (Douma, et al., 2011).

**References**

de Jonge, L.P., Douma, R.D., Heijnen, J.J., and van Gulik, W.M. (2012) Optimization of cold methanol quenching for quantitative metabolomics of *Penicillium chrysogenum*, *Metabolomics* **8**: 727-735.

de Jonge, L.P., Buijs, N.A., ten Pierick, A., Deshmukh, A., Zhao, Z., Kiel, J.A., et al. (2011) Scale-down of penicillin production in *Penicillium chrysogenum*, *Biotechnol J* **6**: 944-958.

Douma, R.D., de Jonge, L.P., Jonker, C.T.H., Seifar, R.M., Heijnen, J.J., and van Gulik, W.M. (2010) Intracellular Metabolite Determination in the Presence of Extracellular Abundance: Application to the Penicillin Biosynthesis Pathway in *Penicillium chrysogenum*, *Biotechnol Bioeng* **107**: 105-115.

Douma, R.D., Batista, J.M., Touw, K.M., Kiel, J.A., Krikken, A.M., Zhao, Z., et al. (2011) Degeneration of penicillin production in ethanol-limited chemostat cultivations of *Penicillium chrysogenum*: A systems biology approach, *Bmc Syst Biol* **5**: 132.

Gonzalez, B., François, J., and Renaud, M. (1997) A rapid and reliable method for metabolite extraction in yeast using boiling buffered ethanol, *Yeast* **13**: 1347-1355.

Mashego, M.R., van Gulik, W.M., Vinke, J.L., and Heijnen, J.J. (2003) Critical evaluation of sampling techniques for residual glucose determination in carbon-limited chemostat culture of *Saccharomyces cerevisiae*, *Biotechnol Bioeng* **83**: 395-399.

Mashego, M.R., Wu, L., Van Dam, J.C., Ras, C., Vinke, J.L., Van Winden, W.A., et al. (2004) MIRACLE: mass isotopomer ratio analysis of U-^13^C-labeled extracts. A new method for accurate quantification of changes in concentrations of intracellular metabolites, *Biotechnol Bioeng* **85**: 620-628.

Seifar, R.M., Deshmukh, A.T., Heijnen, J.J., and van Gulik, W.M. (2012) Determination of δ-[L-α-aminoadipyl]-L-cysteinyl-D-valine in cell extracts of *Penicillium chrysogenum* using ion pair-RP-UPLC-MS/MS, *J Sep Sci* **35**: 225-230.

Wu, L., Mashego, M.R., van Dam, J.C., Proell, A.M., Vinke, J.L., Ras, C., et al. (2005) Quantitative analysis of the microbial metabolome by isotope dilution mass spectrometry using uniformly ^13^C-labeled cell extracts as internal standards, *Anal Biochem* **336**: 164-171.

**Calculation of q-rates in the TCR system**

**μ,** $\text{q}_{\text{CO2}}$ **and** $\text{q}_{\text{Stored carbon}}$ **values**

**For TCR2:**

A large (stored) carbon consumption is observed, which contains glucose consumption (13.68 mmolC/h), central metabolites (19.97 mmolC/h) and storage carbohydrates (77.90 mmolC/h), giving in total an amount of 111.55 mmolC/h. This ends up mostly in biomass growth and $\text{CO}_{\text{2}}$ emission both at 55.78 mmolC/h. Combing the biomass concentration in the vessel (3×5.7/28.05=0.61CmolX), we then obtained μ (91.33 mmol/CmolX/h) and $\text{q}_{\text{CO2}}$ (91.33 mmol/CmolX/h).

**For TCR1:**

A large glucose consumption is observed, which is 132.63 mmolC/h (40×3×5.7/28.05-13.68). This carbon consumption is used for 1) Carbon accumulation (19.97+77.90), about 98 mmolC/h; 2) The remaining carbon consumption is mainly for CO_2_ emission (17.32 mmolC/h) and growth (17.32 mmolC/h). Then we obtained μ (28.39 mmol/CmolX/h) and $\text{q}_{\text{CO2}}$ (28.39 mmol/CmolX/h). Also, we can obtain the formation/consumption rates of the stored carbons (about 98 mmolC/h) which are accumulated in the central metabolites, giving the $\text{q}_{\text{Stored carbon}}$ of 160.66 mmol/CmolX/h.

$\text{q}_{\text{O2}}$ **values**

The $\text{q}_{\text{O2}}$ values were thus estimated using the degree of reduction balances. The thus obtained q-rates were also reconciled under the constraint that the elemental conservation relations were satisfied, using the approach of Verheijen, P.J. (2010).

Verheijen, P.J. (2010) Data reconciliation and error detection, *The metabolic pathway engineering handbook*: 8.1-8.13.

# Supplementary Tables

**Table S1** Average intracellular amounts (in μmol/gDW) of glycolytic, PPP, TCA cycle intermediates measured based on three individual chemostat cultivations within the time range of 100 h to 200 h in the continuous feeding and TCR system. The measurements are based on five independent data points.

| **Metabolites** | **Chemostat**  **Reference** | **TC Systems (6 min)** | |
| --- | --- | --- | --- |
|  |  | **TCR1** | **TCR2** |
| G6P | 2.75±0.26 | 3.32±0.25 | 0.35±0.06 |
| F6P | 0.78±0.08 | 1.67±0.14 | 0.25±0.04 |
| M6P | 1.18±0.10 | 2.10±0.25 | 0.37±0.07 |
| FBP | 0.88±0.11 | 0.94±0.10 | 0.19±0.03 |
| 6PG | 0.06±0.01 | 0.04±0.01 | 0.01±0.00 |
| R5P | 0.94±0.18 | ND | ND |
| 2&3PG | 2.39±0.32 | 2.52±0.14 | 1.35±0.46 |
| PEP | 0.14±0.01 | 0.16±0.01 | 0.12±0.01 |
| Citrate | 1.16±0.11 | 0.72±0.09 | 0.59±0.10 |
| Pyruvate | 0.46±0.05 | 0.82±0.11 | 0.64±0.17 |
| αKG | 1.35±0.25 | 1.18±0.20 | 1.01±0.41 |
| Succinate | 0.27±0.12 | 0.19±0.02 | 0.09±0.02 |
| Fumarate | 1.01±0.07 | 2.79±0.24 | 2.15±0.14 |
| Malate | 1.99±0.18 | 4.48±0.45 | 2.46±0.21 |

**Table S2** Average Storage carbohydrates measured (in μmol/gDW) based on three individual chemostat cultivations within the time range of 100 h to 200 h in the continuous feeding, the IFRs and the TCR system. The measurements are based on five independent data points. In the IFRs, the samples were taken within the famine phase.

| **Metabolites** | **Chemostat**  **Reference** | **IFR** | | | **TCR (6 min)** | |
| --- | --- | --- | --- | --- | --- | --- |
|  |  | **30 s** | **3 min** | **6 min** | **TCR1** | **TCR2** |
| Erythritol | 78.24±21.28 | 41.82±3.51 | 36.99±3.79 | 81.44±11.90 | 69.2±8.97 | 68.99±7.94 |
| Arabitol | 21.34±4.47 | 26.03±0.72 | 9.78±0.36 | 13.06±2.52 | 53.99±9.98 | 38.65±12.8 |
| Mannitol | 380.73±28.54 | 183.50±4.79 | 180.79±10.09 | 82.40±2.97 | 338.70±16.87 | 311.13±9.06 |
| Trehalose | 69.92±5.65 | 95.33±4.89 | 150.69±6.77 | 172.57±2.67 | 38.78±1.93 | 38.37±6.87 |

**Table S3** Average intracellular levels of amino acids measured (in μmol/gDW) based on three individual chemostat cultivations within the time range of 100 h to 200 h in the IFRs and TCR system. Samples in the IFRs were taken in the famine phase and the measurements are based on five independent data points. TCR1: Feed compartment; TCR2: Non-feed compartment.

| **Metabolites** |  | **IFR** | | | | **TCR (6 min)** | | |
| --- | --- | --- | --- | --- | --- | --- | --- | --- |
|  | **Reference** | | **30 s** | **3 min** | **6 min** |  |  |  |
|  |  |  |  |  |  | **TCR1** | **TCR2** | |
| ***Histidine family*** | | | | | |  | |  |
| R5P | 0.99±0.06 | | 1.61±0.16 | 0.67±0.01 | 0.99±0.06 | ND | | ND |
| Histidine | 0.83±0.06 | | 3.40±0.08 | 5.2±0.40 | 1.56±0.12 | 2.28±0.38 | | 1.80±0.20 |
| ***Serine family*** | | | | | |  | |  |
| 2&3PG | 2.49±0.09 | | 2.07±0.05 | 1.15±0.04 | 1.06±0.10 | 2.52±0.14 | | 1.35±0.46 |
| Cysteine | 1.37±0.02 | | 0.47±0.01 | 0.70±0.02 | 0.48±0.03 | 1.03±0.26 | | 0.97±0.11 |
| Serine | 13.44±0.22 | | 13.19±0.21 | 12.98±0.19 | 12.32±0.41 | 12.80±0.50 | | 12.67±1.54 |
| Glycine | 6.72±0.16 | | 5.87±0.10 | 6.24±0.16 | 6.25±0.08 | 8.57±0.41 | | 9.14±0.95 |
| ***Aromatic family*** | | | | | |  | |  |
| PEP | 0.15±0.01 | | 0.30±0.01 | 0.11±0.00 | 0.82±0.11 | 0.31±0.05 | | 0.85±0.16 |
| Phenylalanine | 0.22±0.01 | | 0.27±0.02 | 0.31±0.01 | 0.26±0.01 | 0.32±0.02 | | 0.32±0.09 |
| Tyrosine | 0.32±0.04 | | ND | ND | ND | ND | | ND |
| ***Pyruvate family*** | | | | | |  | |  |
| Pyruvate | 0.51±0.02 | | 0.87±0.03 | 0.64±0.01 | 5.003±0.13 | 0.82±0.11 | | 0.64±0.17 |
| Alanine | 49.88±0.93 | | 74.30±3.63 | 80.04±0.90 | 76.41±0.97 | 65.53±4.59 | | 56.29±5.51 |
| Valine | 3.23±0.05 | | 4.02±0.24 | 4.86±0.07 | 4.97±0.16 | 4.28±0.17 | | 3.62±0.28 |
| Leucine | 1.12±0.04 | | 1.34±0.02 | 1.12±0.03 | 1.32±0.03 | 1.21±0.08 | | 1.31±0.14 |
| ***Aspartate family*** | | | | | |  | |  |
| Malate | 2.05±0.05 | | 5.12±0.12 | 6.25±0.19 | 3.35±0.48 | 4.48±0.45 | | 2.46±0.21 |
| Aspartate | 37.92±1.13 | | 41.69±2.26 | 54.67±1.16 | 47.65±0.86 | 39.69±1.70 | | 50.35±2.15 |
| Asparagine | 3.72±0.10 | | 0.71±0.03 | 1.04±0.02 | 1.01±0.02 | 1.40±0.08 | | 1.44±0.06 |
| Methionine | 0.32±0.01 | | 0.29±0.01 | 0.32±0.01 | 0.27±0.01 | NA | | NA |
| Threonine | 12.28±0.16 | | 12.73±0.22 | 14.98±0.18 | 12.53±0.29 | NA | | NA |
| Isoleucine | 0.46±0.02 | | 0.69±0.06 | 0.68±0.02 | 0.87±0.03 | 0.71±0.07 | | 0.77±0.08 |
| ***Glutamate family*** | | | | | |  | |  |
| αKG | 1.47±0.07 | | 1.34±0.04 | 0.87±0.02 | 0.65±0.08 | 1.18±0.20   \| 2.460804126 \| \| --- \| \| 0.207853228 \| | | 1.01±0.41 |
| Glutamate | 115.24±4.41 | | 107.14±3.05 | 123.49±3.23 | 101.90±8.65 | 90.94±6.78 | | 85.77±5.07 |
| Glutamine | 47.54±1.05 | | 54.52±1.40 | 47.33±0.58 | 44.32±2.98 | 44.69±3.19 | | 43.96±2.60 |
| Ornithine | ND | | ND | ND | ND | ND | | ND |
| Proline | 1.60±0.04 | | 2.05±0.03 | 2.20±0.05 | 1.95±0.09 | 1.80±0.14 | | 1.79±0.17 |
| αAAA | 1.62±0.05 | | 1.42±0.06 | 0.97±0.02 | 1.00±0.11 | 0.76±0.05 | | 0.72±0.06 |
| Lysine | 1.60±0.02 | | 1.49±0.09 | 0.77±0.07 | 1.01±0.14 | 5.11±1.08 | | 5.73±1.71 |

# Supplementary Figures

**
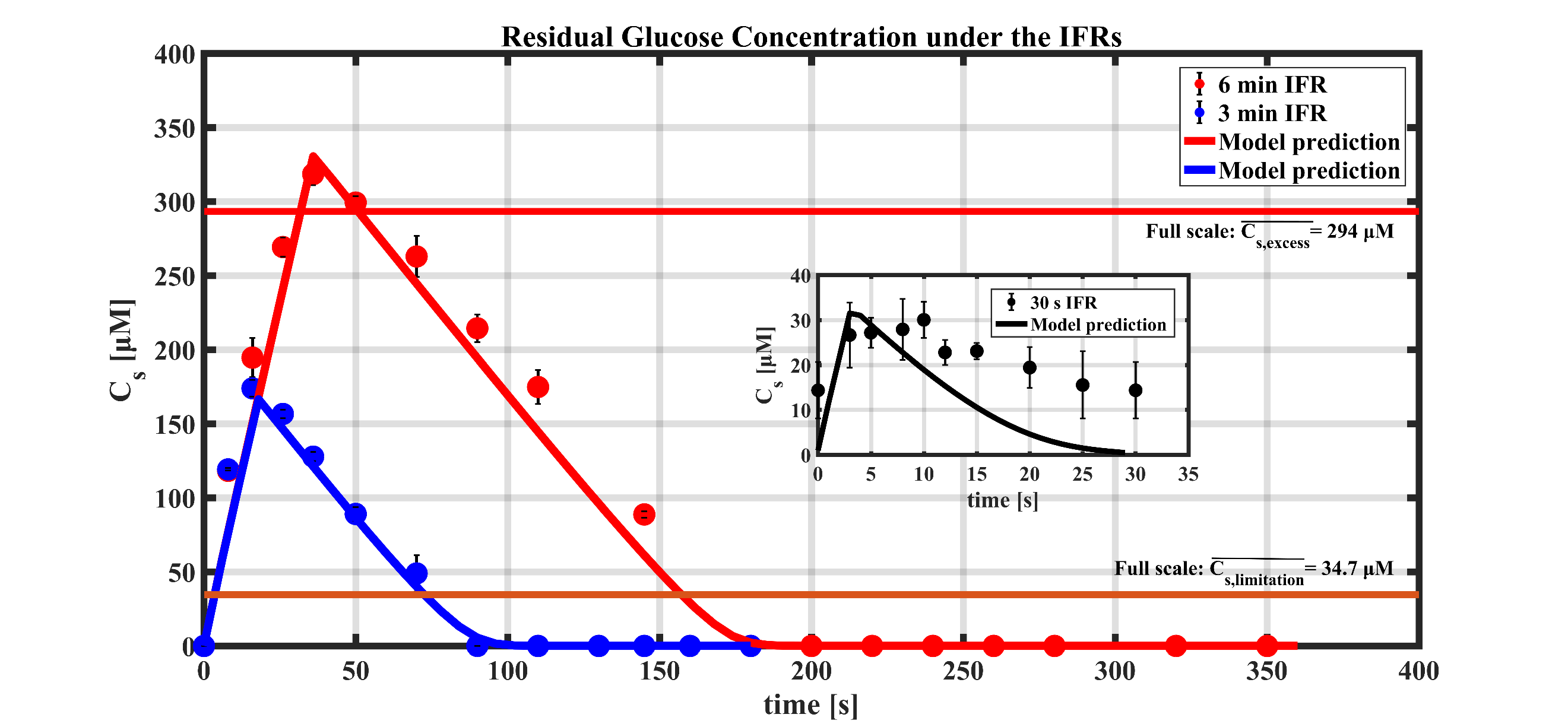
**

**Fig.S1** Residual glucose concentration ($\text{C}_{\text{s}}$) profiles in the IFRs.

**
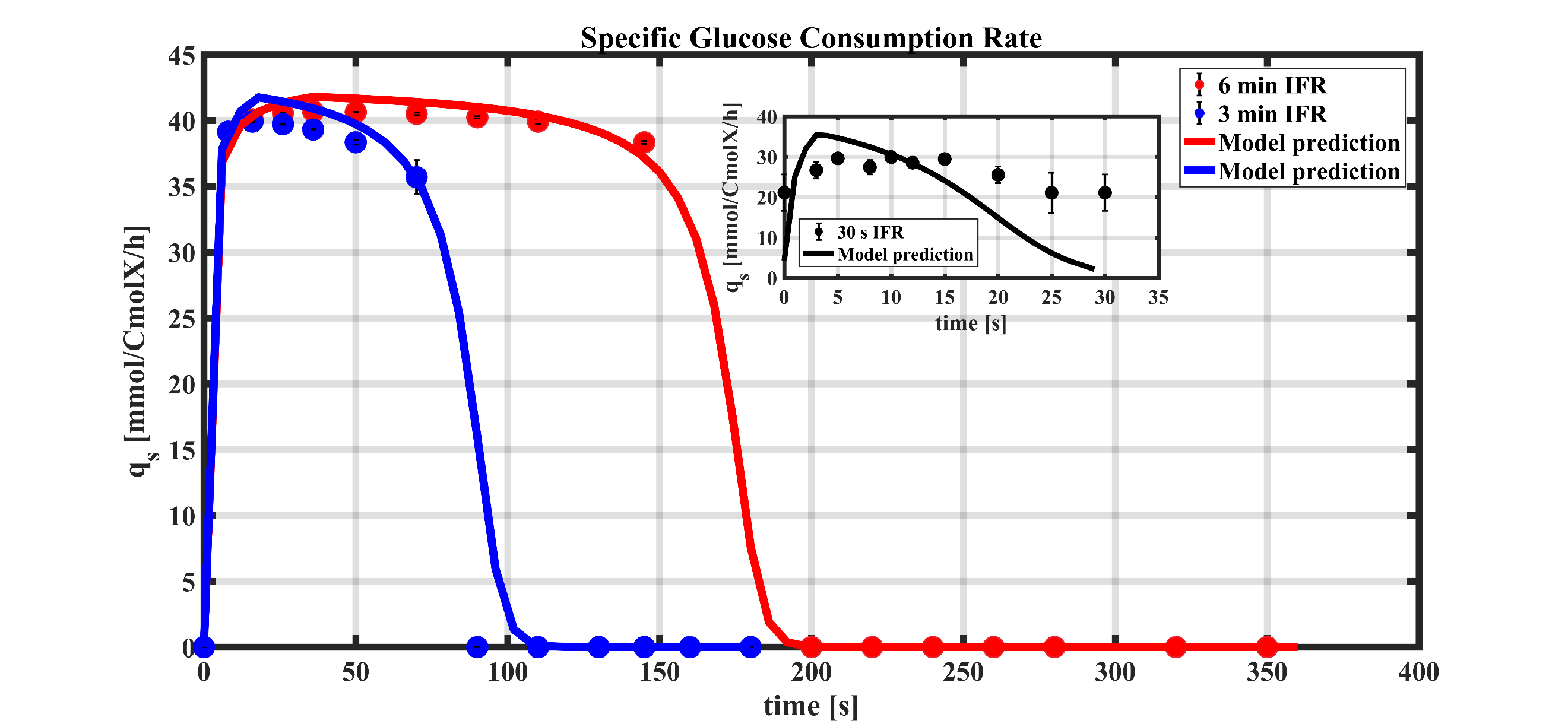
**

**Fig.S2** Specific glucose uptake rate ($\text{q}_{\text{s}}$). Red, blue and black dots represent the results of 6 min, 3 min and 30 s IFRs, respectively. Solid line represents the model prediction. All data points are based on three individual feeding cycles after five residence times of chemostat cultures.

**
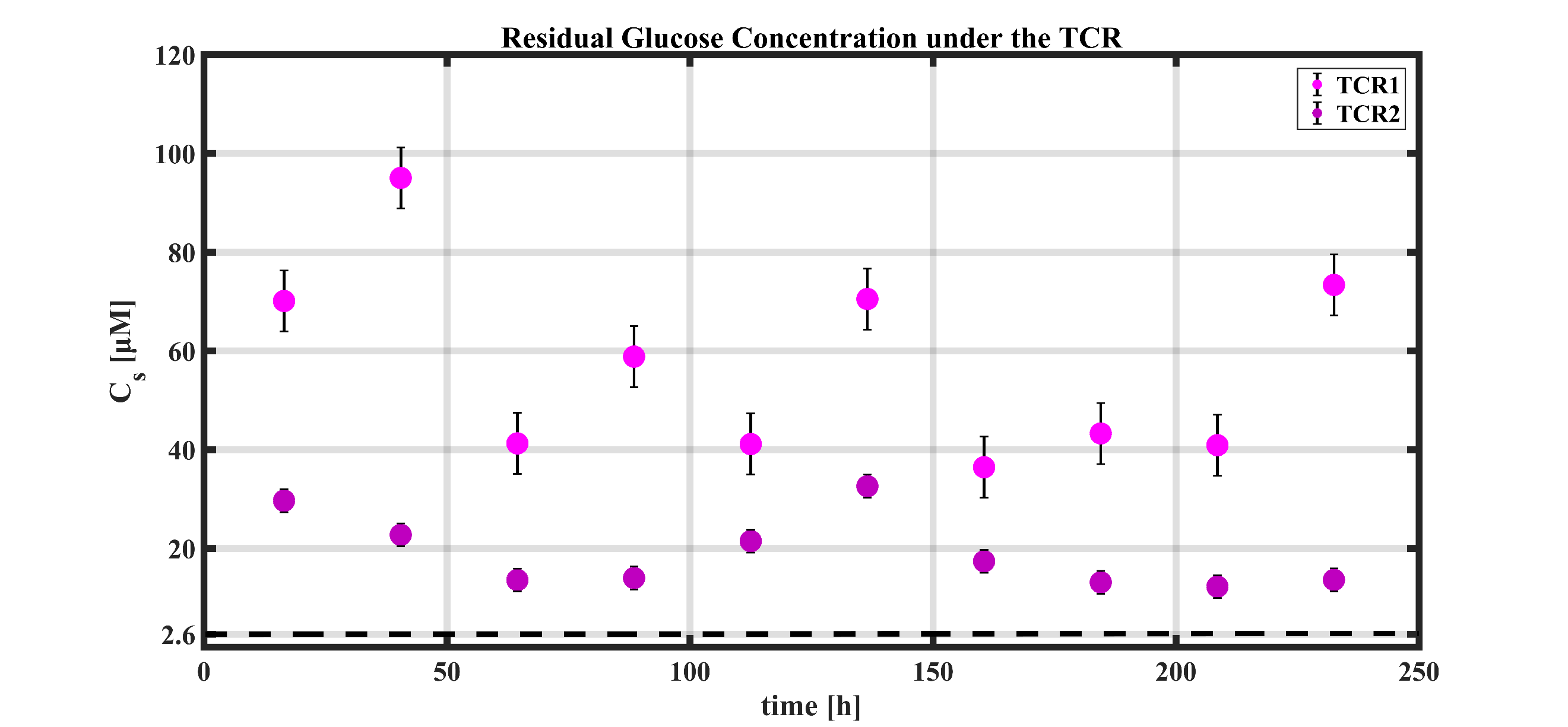
**

**Fig.S3** Extracellular $\text{C}_{\text{s}}$ values as a function of time in the TCR system. The dashed line represents the mean value in the reference chemostat cultures.

**
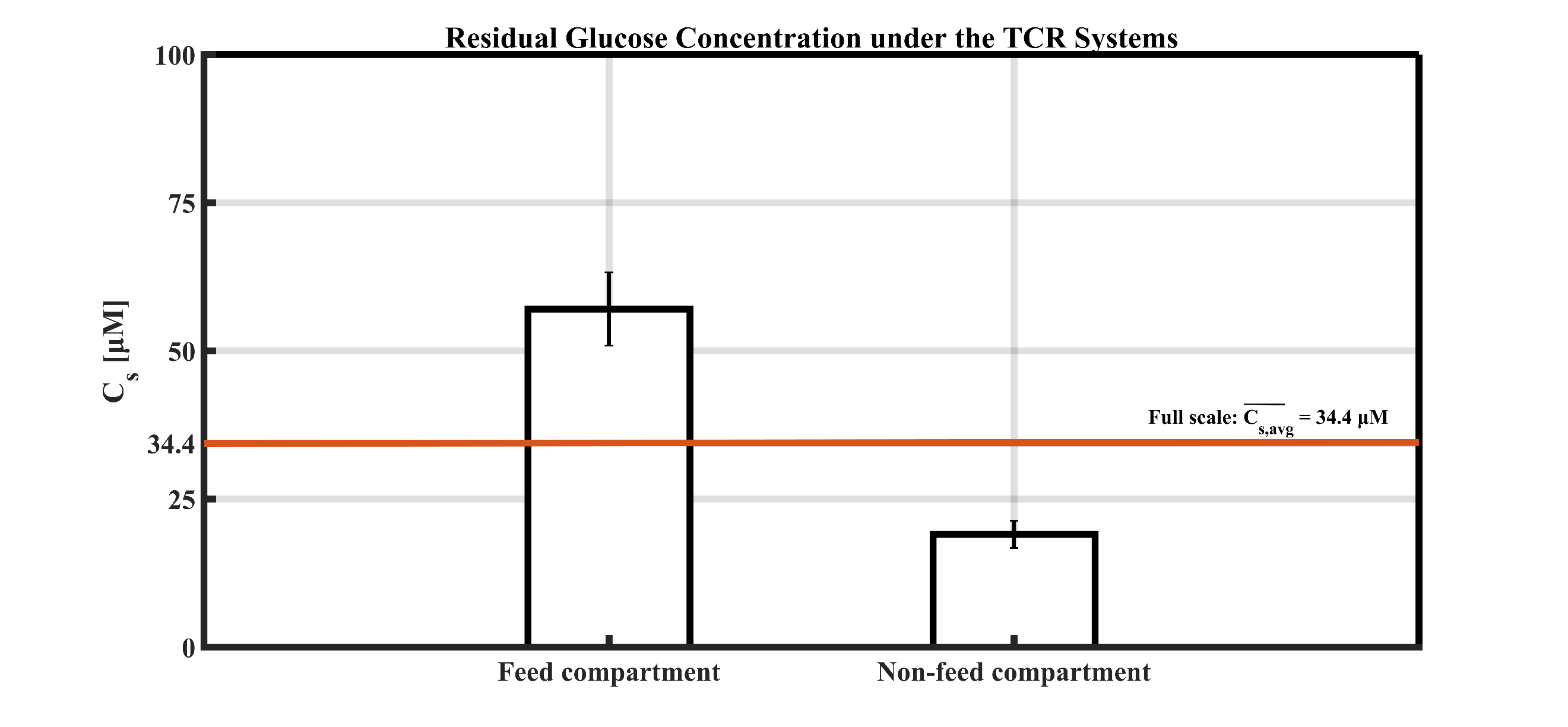
**

**Fig.S4** Average residual glucose concentrations in the TCR system are based on 10 independent data points.

**
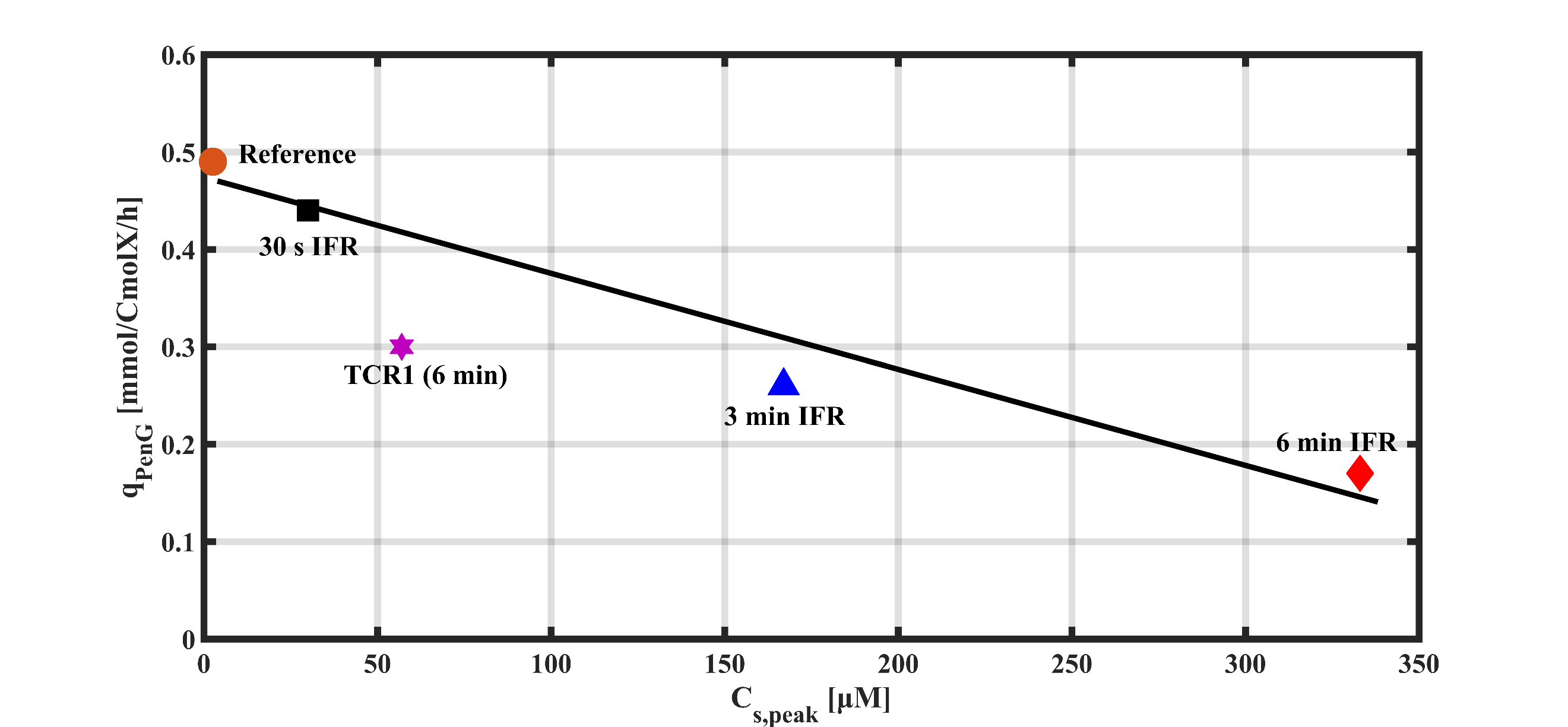
**

**Fig.S5** $\text{q}_{\text{PenG}}$ against $\text{C}_{\text{s, peak}}$.

**
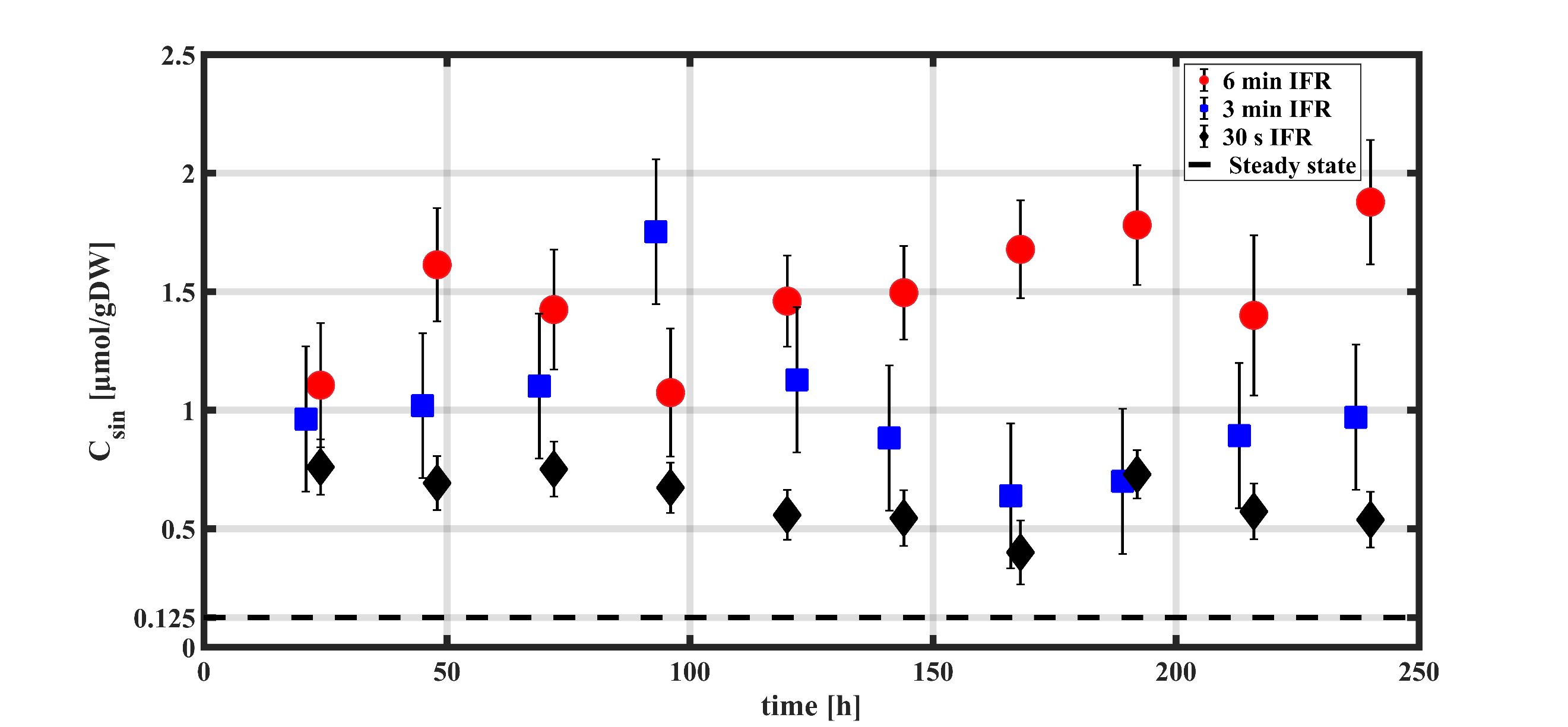
**

**Fig.S6** Intracellular glucose levels as function of the culture age in the IFRs. The dashed line represents the mean value in the reference chemostat cultures.

**
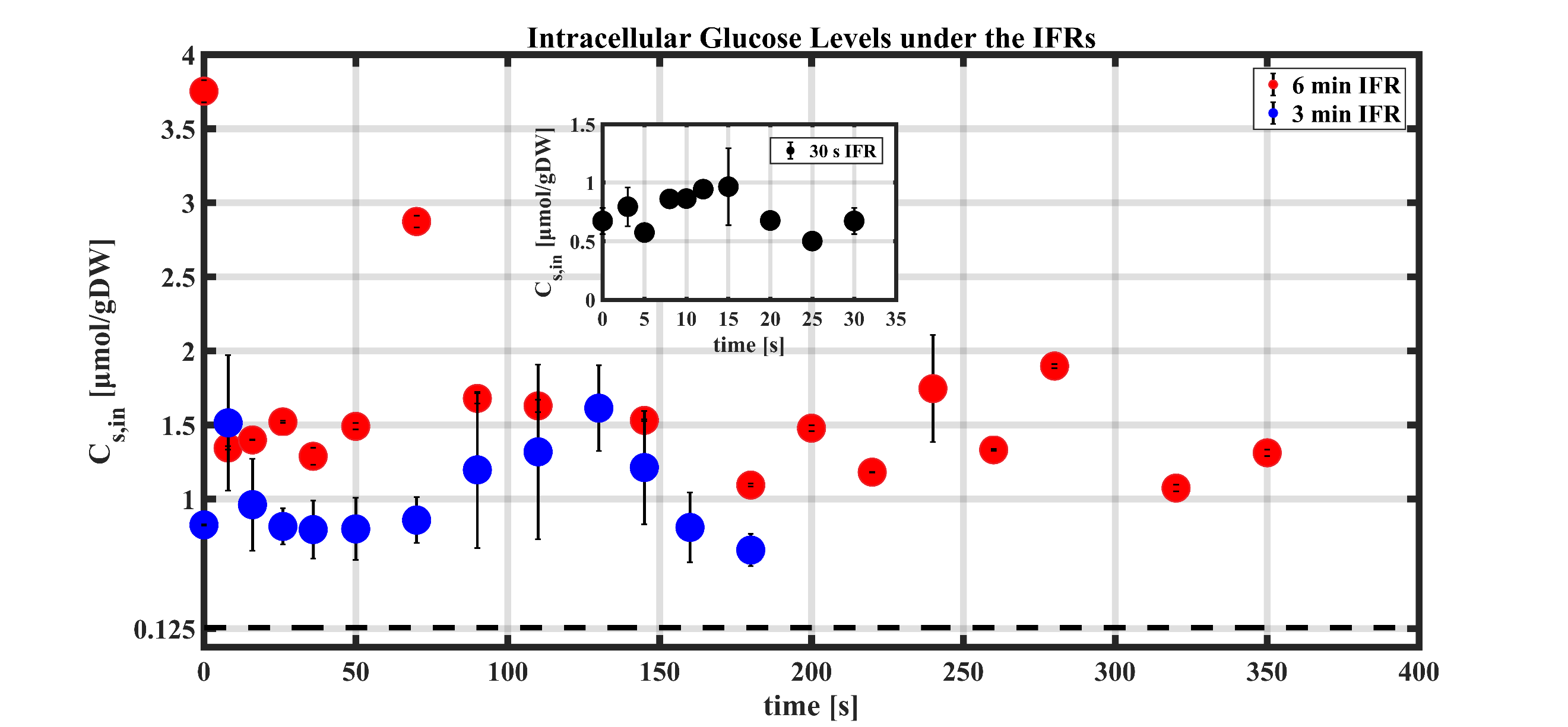
**

**Fig.S7** Intracellular glucose levels over a complete feed cycle in the IFRs. All data points in the IFRs are based on three individual feeding cycles after five residence times of chemostat cultures. The dashed line represents the mean value in the reference chemostat cultures.

**
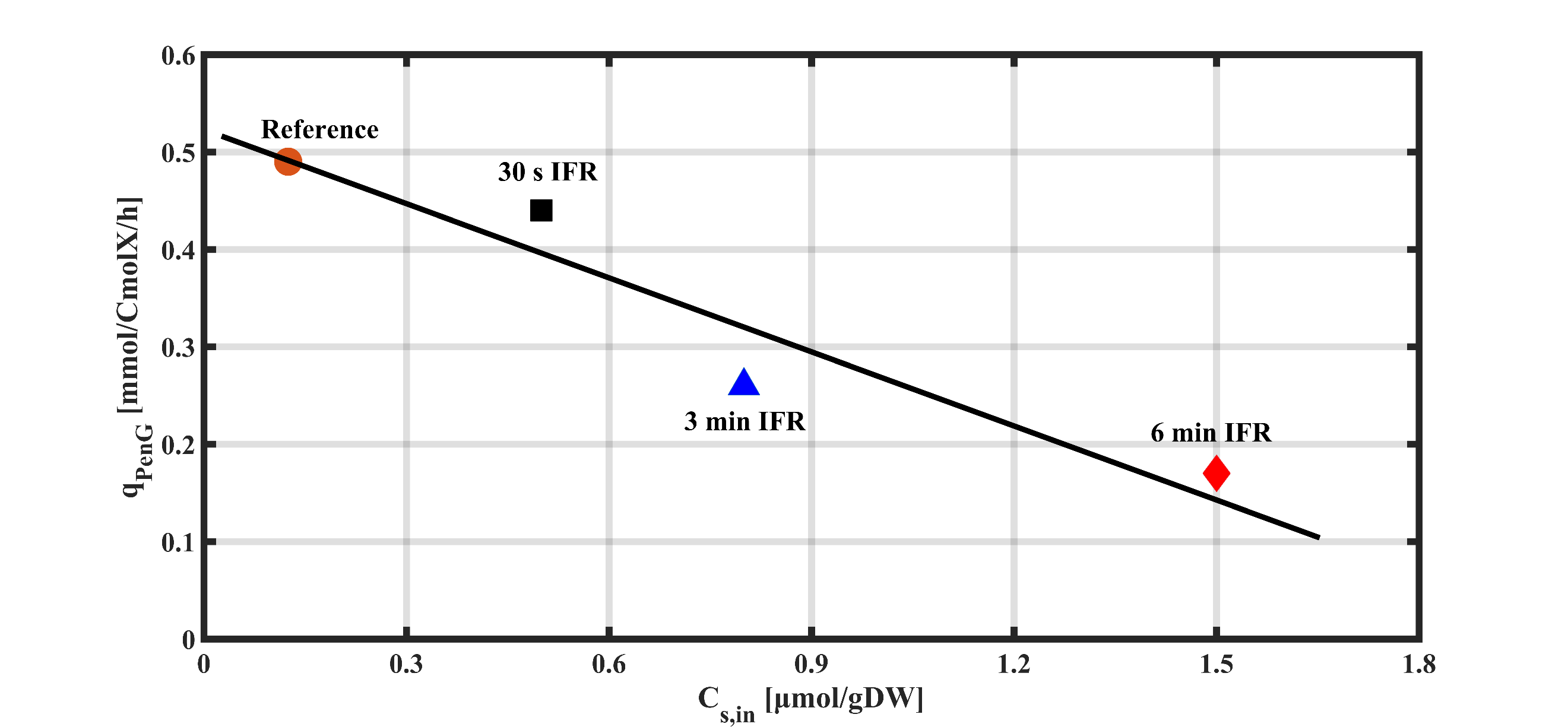
**

**Fig.S8** $\text{q}_{\text{PenG}}$ against $\text{C}_{\text{s, in}}$ in the IFRs.

**
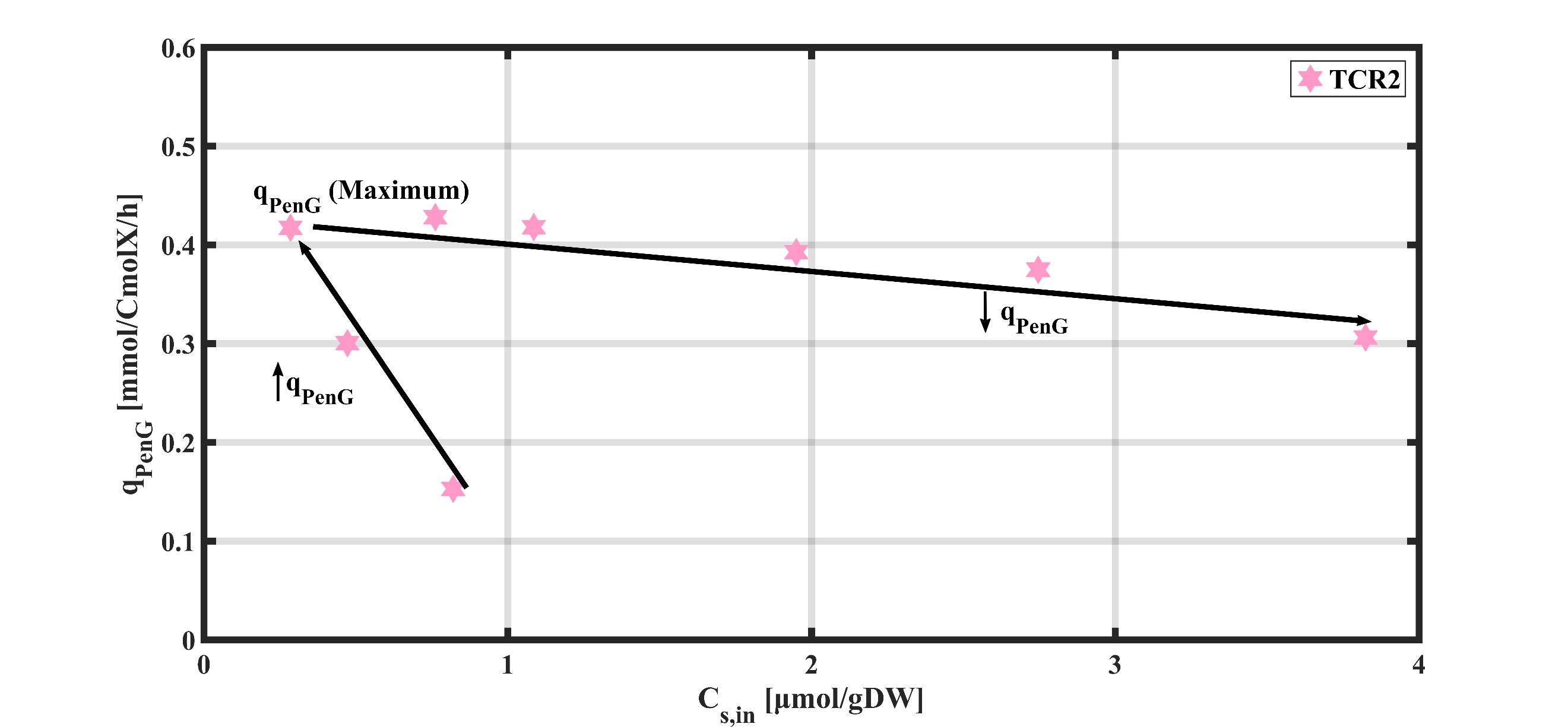
**

**Fig.S9** $\text{q}_{\text{PenG}}$ against $\text{C}_{\text{s, in}}$ in the TCR2.

**
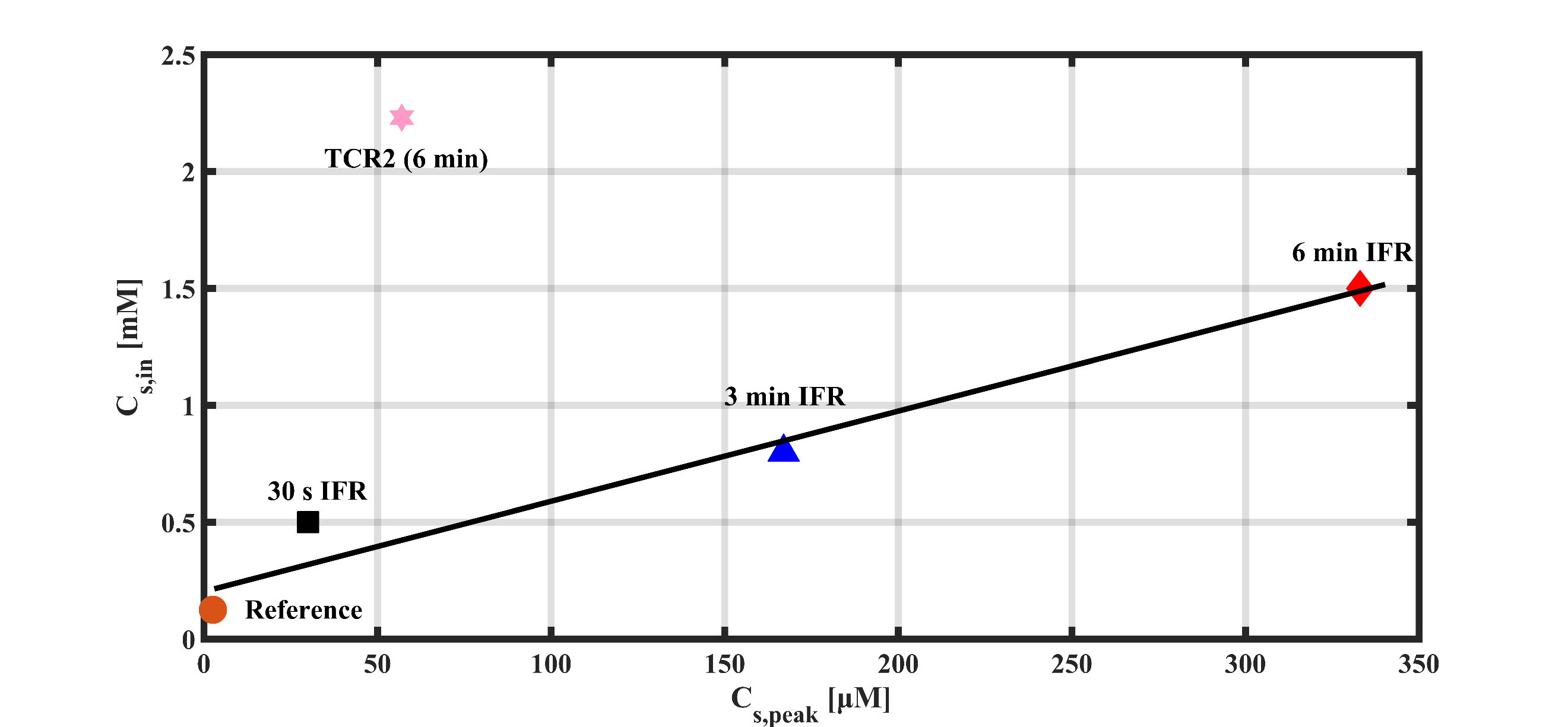
**

**Fig.S10** $\text{C}_{\text{s, in}}$ against $\text{C}_{\text{s, peak}}$.

**
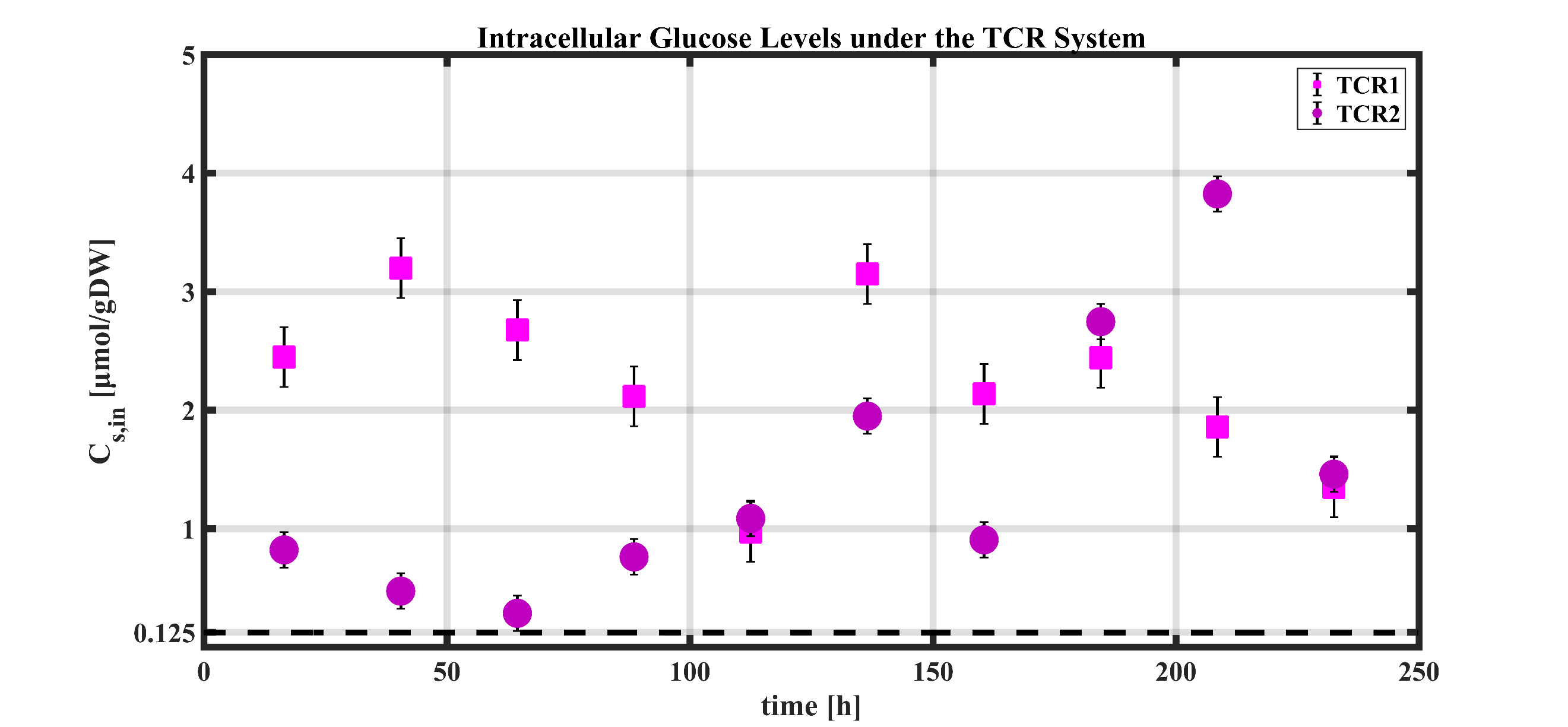
**

**Fig.S11** Intracellular glucose levels as function of the culture age in the TCR. The dashed line represents the mean value in the reference chemostat cultures.

**
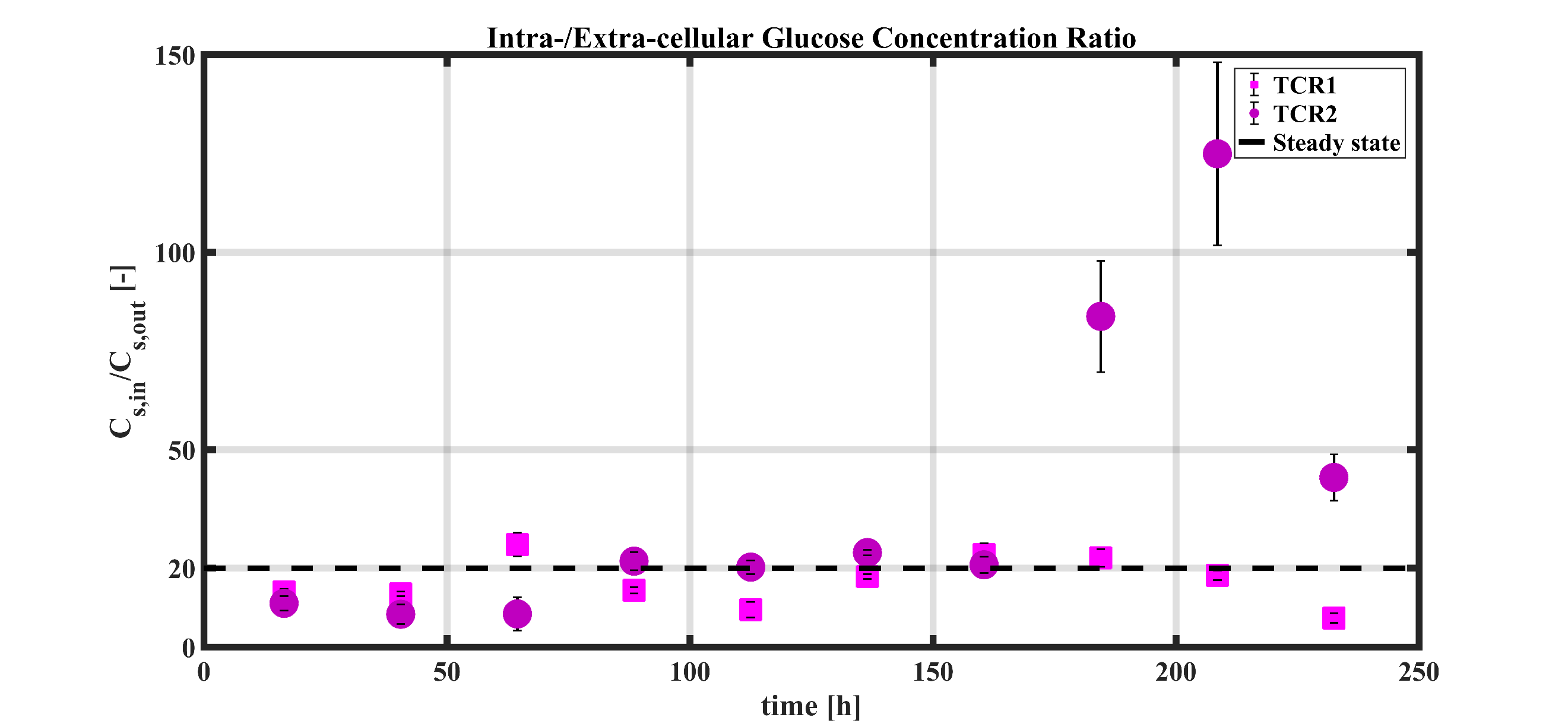
**

**Fig.S12** The intra/extra-cellular glucose concentration ratio in the TCR system, where 2.5 ml/gDW is assumed for the conversion.


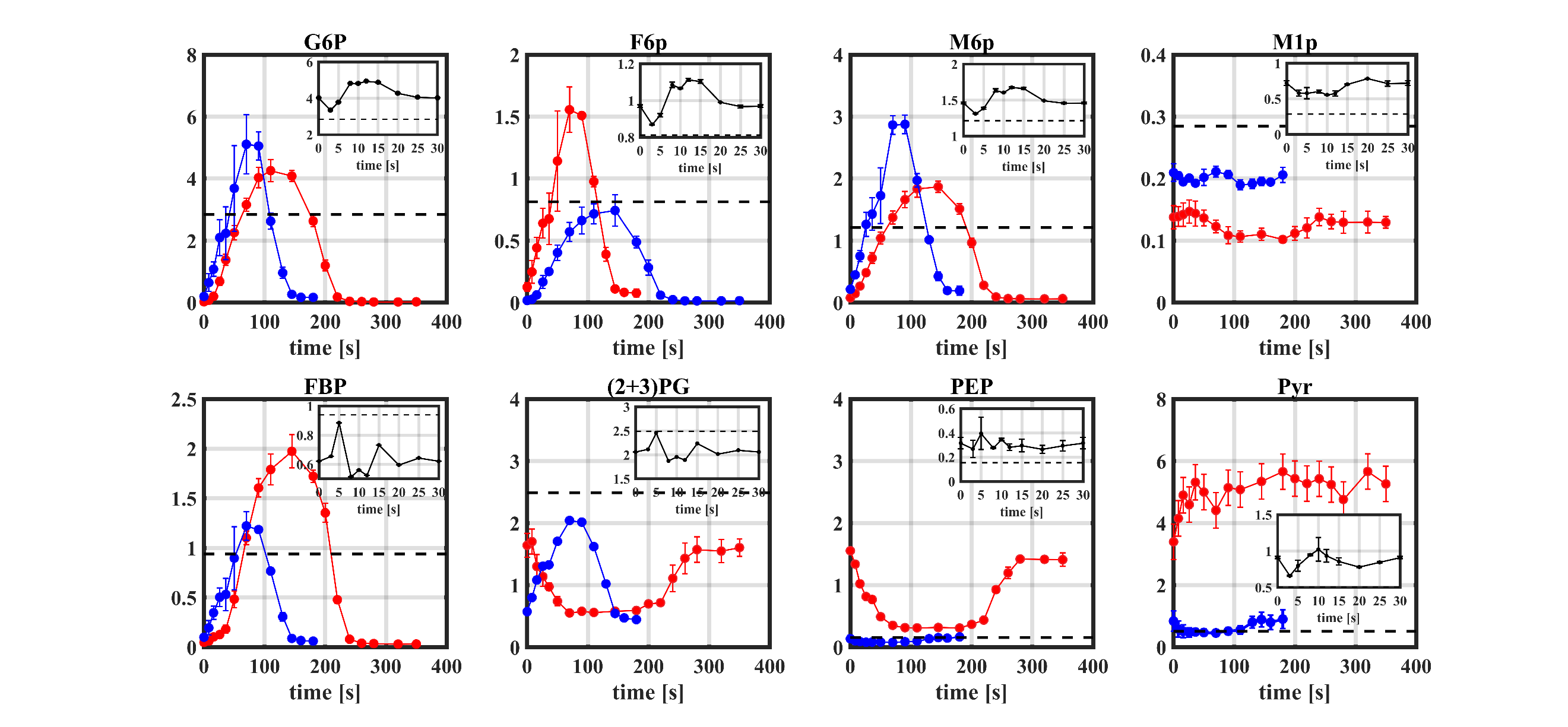


**Fig.S13** ***Metabolites in Glycolysis***


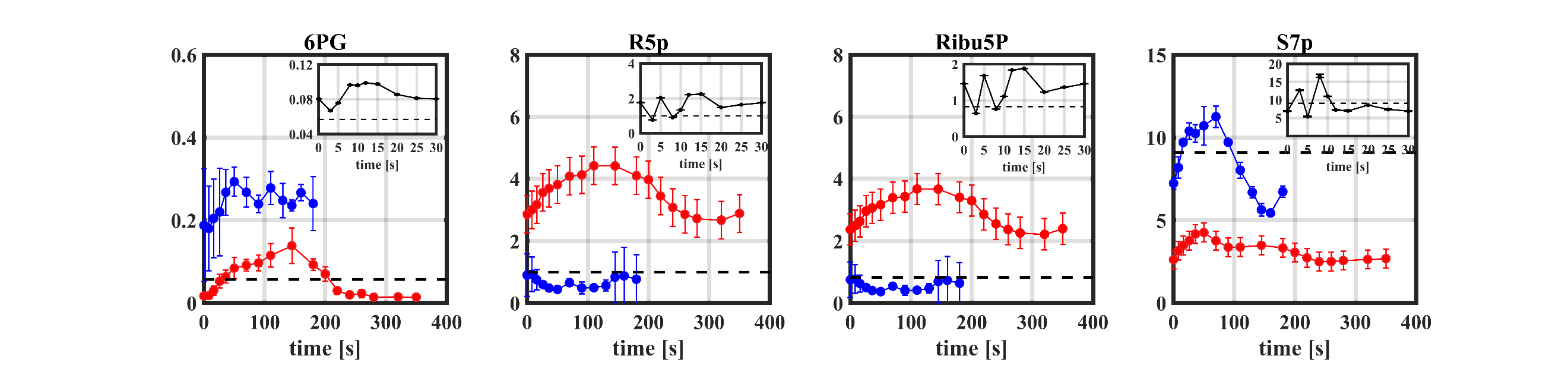


**Fig.S13 *Metabolites in PPP***

**
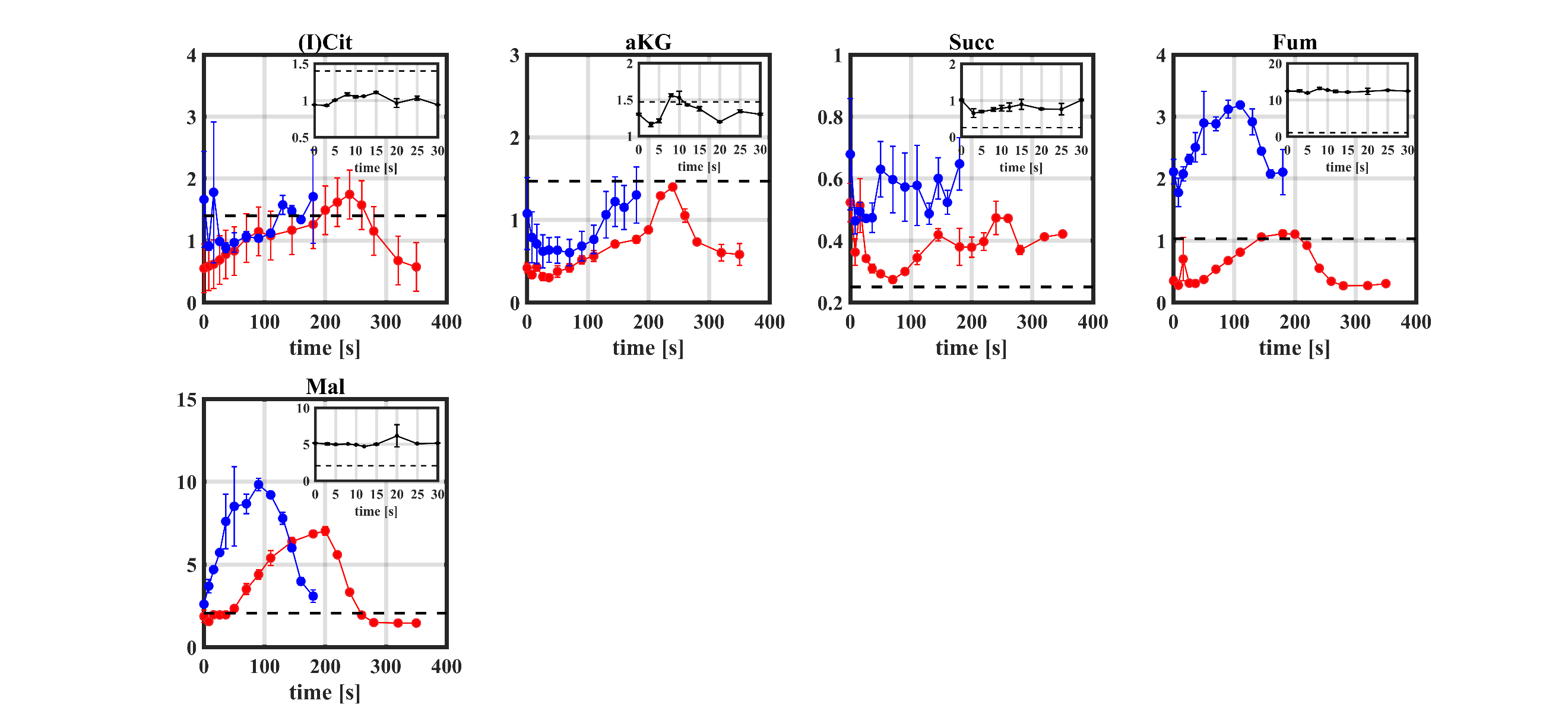
**

**Fig.S13** ***Metabolites in TCA Cycle***


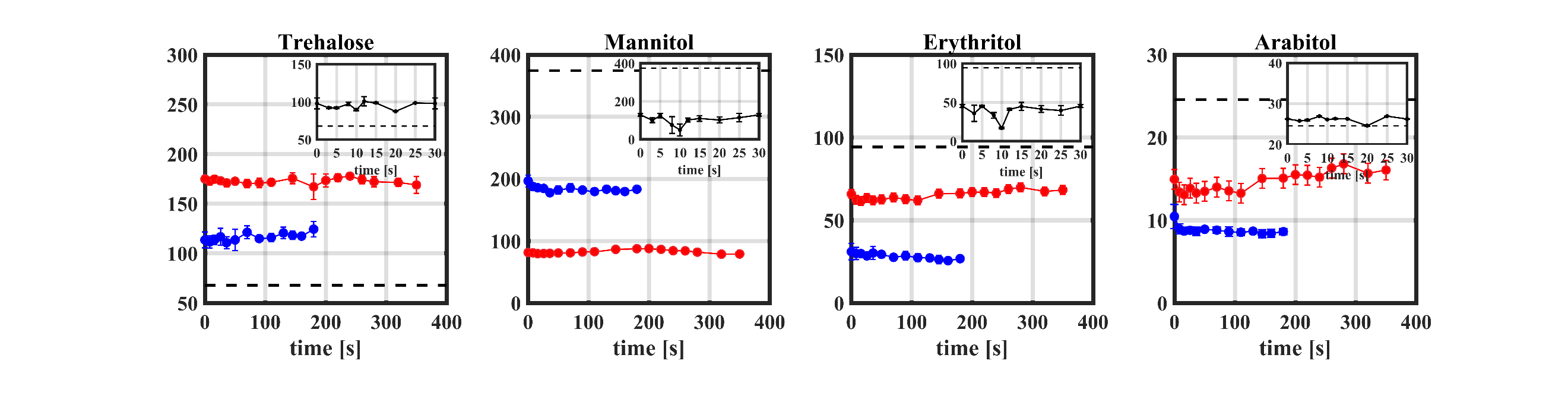


**Fig.S13** ***Metabolites in Storage Pools***

**Fig.S13** Concentration measurements of metabolites (in μmol/gDW) in the glycolysis, PPP, TCA cycle and storage pools. The dashed line represents the average measurements in the reference chemostat cultures. Red, blue and black dots represent the results of 6 min, 3 min and 30 s IFRs, respectively.


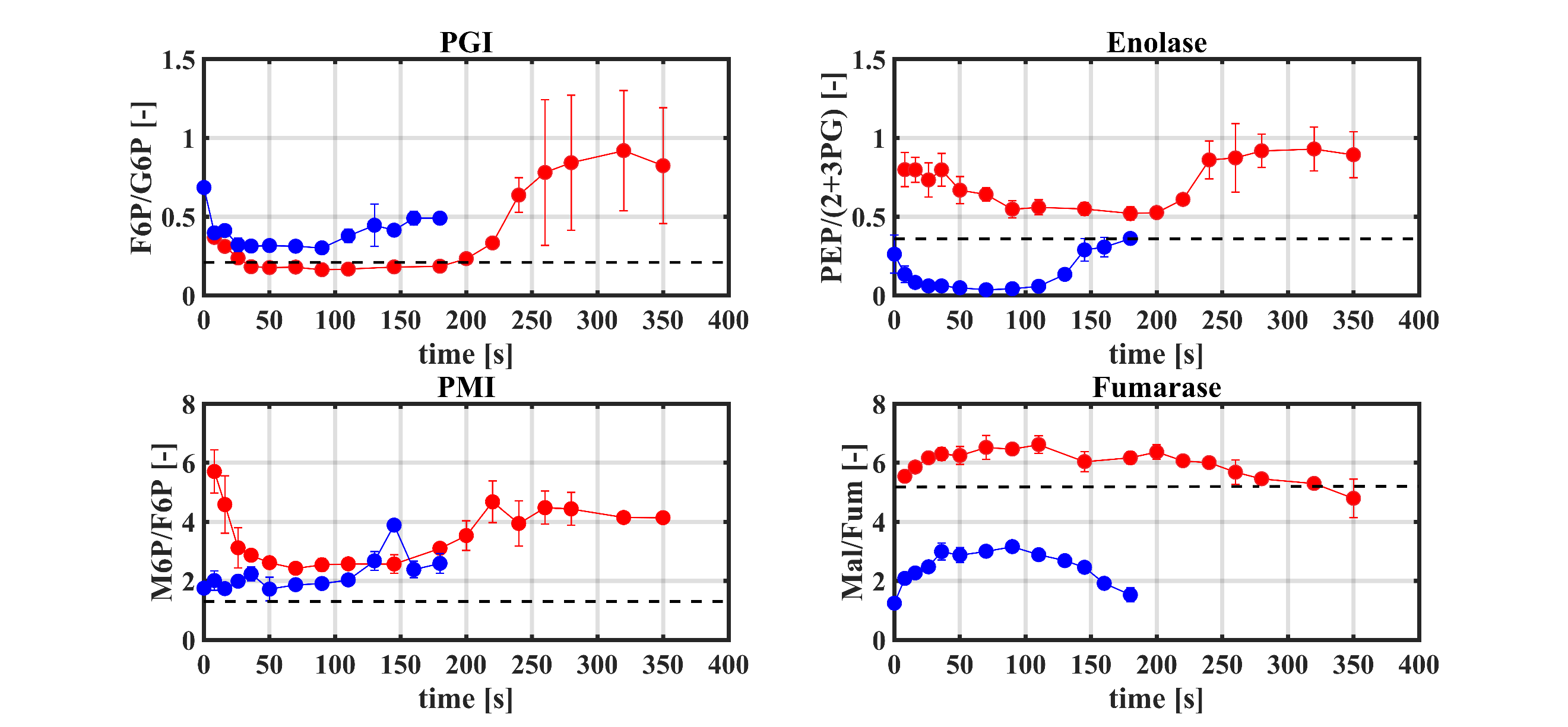


**Fig.S14** Mass action ratios for phosphoglucose isomerase (PGI), enolase, mannose-6-phosphate isomerase (PMI) and fumarase. Red and blue dots represent the results of 6 min and 3 min IFRs, respectively. The dashed lines represent the equilibrium values.


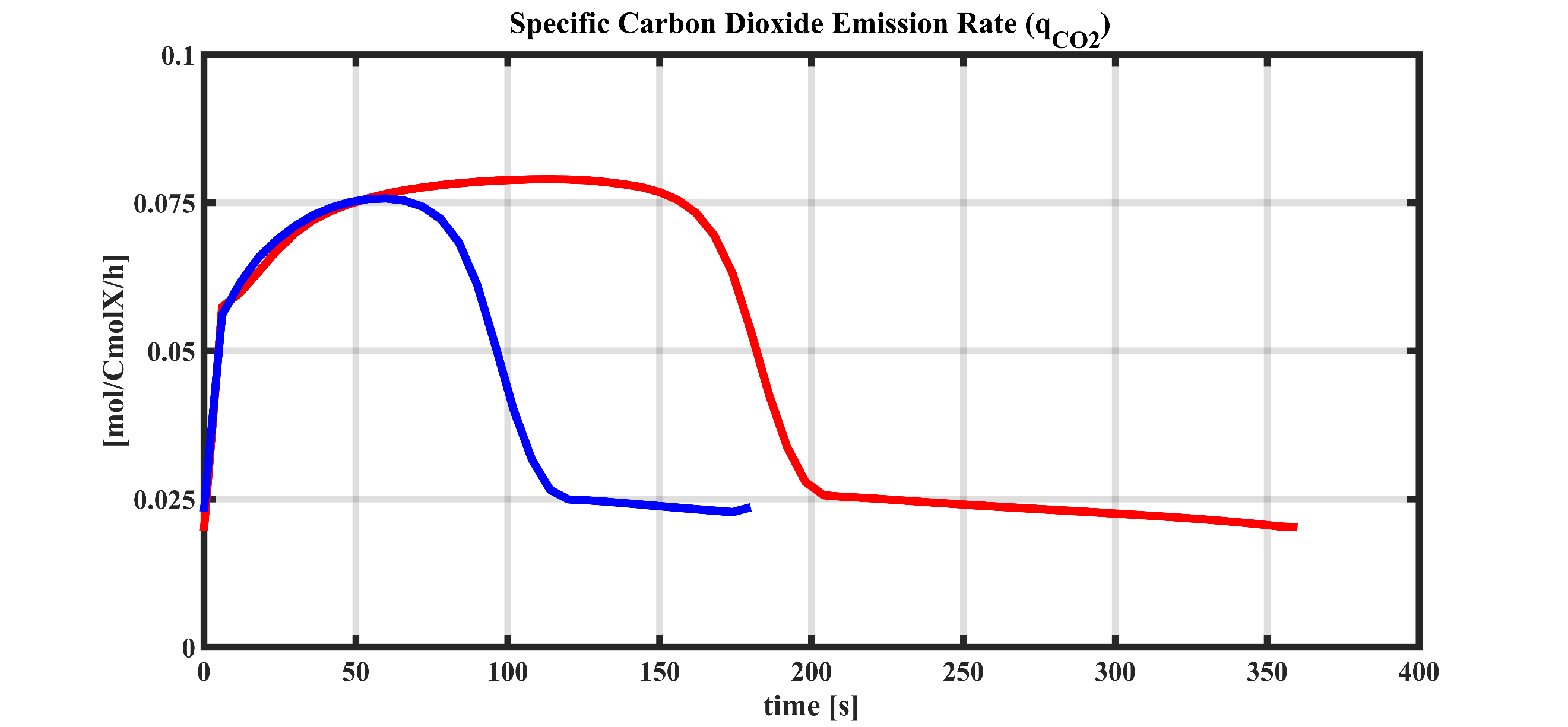


**Fig.S15** The biomass specific carbon emission rates within a complete feeding cycle predicted by the 9-pool model (Tang et al. 2017).


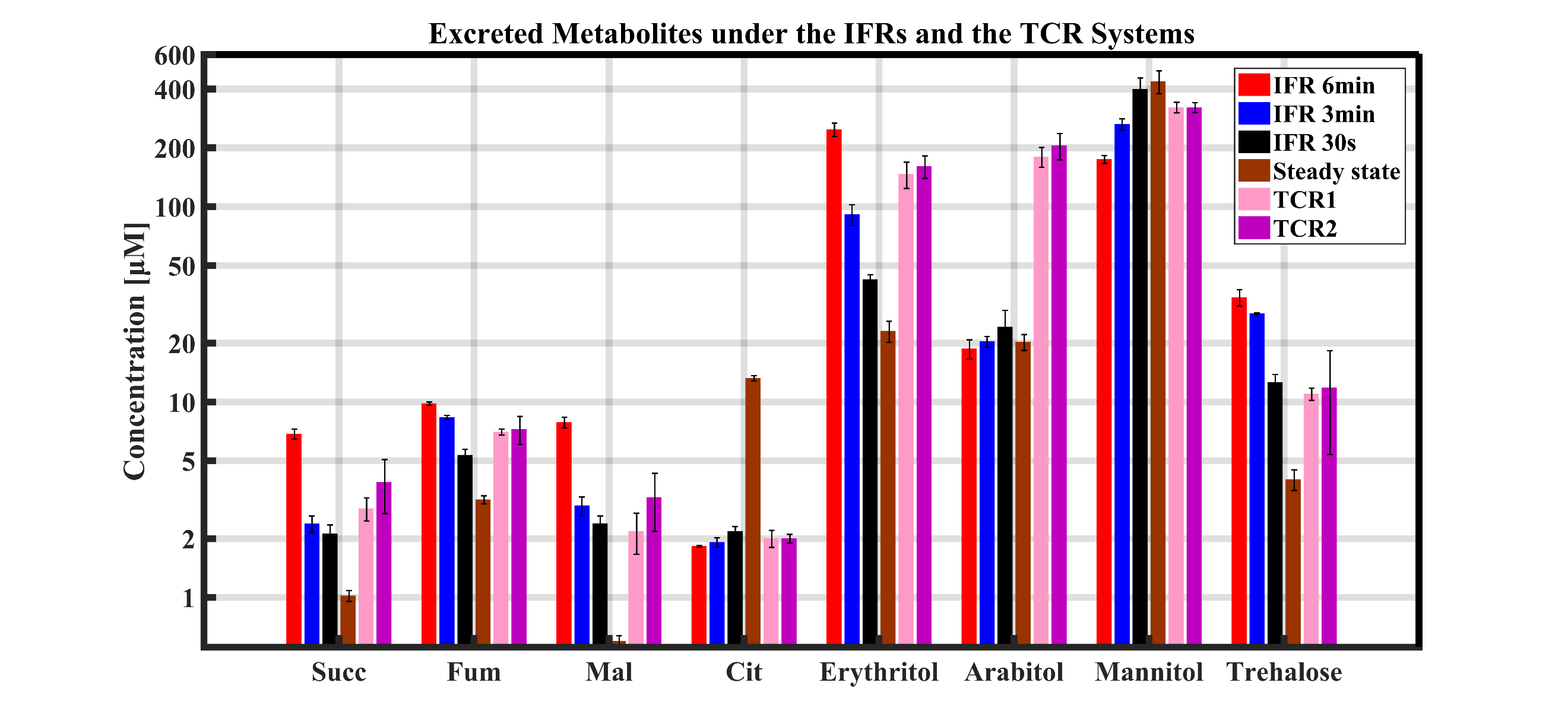


**Fig.S16** Extracellular metabolites in the IFRs and the TCR system. The data are average values of five independent data points from 100 h to 200 h of chemostat cultures. In the IFRs, the samples were taken within the famine phase.

# A 9-pool Metabolic Structured Kinetic Model

A 9-pool metabolic structured kinetic model was used to capture the off-gas $\text{CO}_{\text{2}}$dynamics in the experiments of IFRs. As shown in Fig.S17, this model (Tang et al. 2017) contains five compound pools, namely, glycolytic intermediate pool (Glyc), amino acid pool (AA), carbon carbohydrate pool (Csto), PAA pool and ATP pool. The formula of each pool was in detail defined as reported by Tang et al. 2017. The dry cell element formula was defined as CH_1.79_O_0.59_N_0.16_ (MW_DCW, 28.05 g/CmolX), according to element analysis result by van Gulik (unpublished data).

Fig.S17 Overview of the 9-pool model for *P. chrysogenum*. Carbon metabolism is shown in black, energy metabolism in red, penicillin production in green, storage carbon cycling in blue. [reprinted from (Tang et al. 2017) with permission]

10 reactions were used to describe carbon metabolism, energy metabolism, penicillin production and carbon storage/release (Table S4). Table S5 shows that kinetic models were incorporated into each reaction, the majority of which were based on saturation-type kinetics. Among these dynamic models, the dynamic model for penicillin production was derived from a previous one by Douma et al. (2010). The parameters in the kinetic models can be found in Table S6. Combining compound balances for liquid phase (broth) and gas phase (Tang et al. 2017), dynamic pool profiles, q-rate profiles and off-gas profiles during the feast-famine cycles can be in silico simulated in Matlab R2016b.

Tang, WJ., Deshmukh, A.T., Haringa, C., Wang, G., van Gulik, W., van Winden, W., et al. (2017) A 9-pool metabolic structured kinetic model describing days to seconds dynamics of growth and product formation by *Penicillium chrysogenum*, *Biotechnol Bioeng* **114**: 1733-1743.

Douma, R.D., Verheijen, P.J., de Laat, W.T., Heijnen, J.J., and van Gulik, W.M. (2010) Dynamic gene expression regulation model for growth and penicillin production in Penicillium chrysogenum, Biotechnol Bioeng **106**: 608-618.

Table S4. Complete stoichiometric matrix of the metabolically structured kinetic model (Tang et al. 2017)

|  |  | Carbon metabolism | | | Energy metabolism | | PenG Production | | | Carbohydrate Storage/release | |
| --- | --- | --- | --- | --- | --- | --- | --- | --- | --- | --- | --- |
|  |  | Glc uptake | AA synthesis | Growth | Oxidation | maintenance | PAA import | PAA export | PenG synthesis | Storage | Release |
|  |  | *v*_1.1_ | *v*_1.2_ | *v*_1.3_ | *v*_2.1_ | *v*_2.2_ | *v*_3.1_ | *v*_3.2_ | *v*_3.3_ | *v*_4.1_ | *v*_4.2_ |
| Intracellular | Glyc | 6 | -1 | -0.5775 | -1 | 0 | 0 | 0 | -4.8125 | -1.07 | 1 |
|  | AA | 0 | 1 | -0.5 | 0 | 0 | 0 | 0 | -6.25 | 0 | 0 |
|  | Sto | 0 | 0 | 0 | 0 | 0 | 0 | 0 | 0 | 1 | -1 |
|  | ATP | -2 | -0.6510 | -1.0370 | 4.4300 | -1 | 0 | -2 | -8 | -0.1667 | -0.1667 |
|  | PAA | 0 | 0 | 0 | 0 | 0 | 1 | -1 | -1 | 0 | 0 |
| Extracellular | Glc | -1 | 0 | 0 | 0 | 0 | 0 | 0 | 0 | 0 | 0 |
|  | X | 0 | 0 | 1 | 0 | 0 | 0 | 0 | 0 | 0 | 0 |
|  | PenG | 0 | 0 | 0 | 0 | 0 | 0 | 0 | 1 | 0 | 0 |
|  | PAA | 0 | 0 | 0 | 0 | 0 | -1 | 1 | 0 | 0 | 0 |
|  | O_2_ | 0 | -0.09 | 0 | -1 | 0 | 0 | 0 | -1 | 0 | -0.07 |
|  | CO_2_ | 0 | 0 | 0.0775 | 1 | 0 | 0 | 0 | 3.0625 | 0.07 | 0 |
|  | H^+^ | 0 | 0.32 | 0 | 0 | 0 | 0 | 0 | -2 | 0 | 0 |
|  | SO_4_^2-^ | 0 | 0 | 0 | 0 | 0 | 0 | 0 | -2 | 0 | 0 |
|  | H_2_O | 0 | 0.48 | 0.1825 | 1 | 0 | 0 | 0 | 7.0625 | -0.05 | 0.12 |
|  | NH_4_^+^ | 0 | -0.32 | 0 | 0 | 0 | 0 | 0 | 0 | 0 | 0 |

Table S5. Kinetics used in the metabolically structured kinetic model* (Tang et al. 2017)

| Category | Kinetics | Eq. |
| --- | --- | --- |
| Carbon uptake | $\frac{dX_{E,1.1}}{dt}=q_{E,1.1,\max}\frac{\left[\left(\mu+\delta_{0}\right)/k_{1.1}\right]^{5}}{\left[\left(\mu+\delta_{0}\right)/k_{1.1}\right]^{5}+1}-\left(\mu+k_{dE,1.1}\right)X_{E,1.1}$ | S1 |
|  | $v_{1.1}=k_{E,1.1}\cdot X_{E,1.1}\cdot\frac{C_{S}}{C_{S}+K_{S,1.1}}$ | S2 |
| Amino acid synthesis | $v_{1.2}=v_{1.2,\max}\cdot\frac{X_{Glyc}^{2}}{K_{Glyc,1.2}^{2}+X_{Glyc}^{2}}\cdot\frac{K_{i,AA,1.2}^{2}}{K_{i,AA,1.2}^{2}+X_{AA}^{2}}\cdot\frac{X_{ATP}^{3}}{K_{ATP,1.2}^{3}+X_{ATP}^{3}}$ | S3 |
| Growth | $v_{1.3}=v_{1.3,\max}\cdot\frac{X_{Glyc}^{2}}{K_{Glyc,1.3}^{2}+X_{Glyc}^{2}}\cdot\frac{X_{AA}^{2}}{K_{i,AA,1.3}^{2}+X_{AA}^{2}}\cdot\frac{X_{ATP}^{3}}{K_{ATP,1.3}^{3}+X_{ATP}^{3}}$ | S4 |
| Energy production | $v_{2.1}=v_{2.1,\max}\cdot\frac{X_{Glyc}^{3}}{K_{Glyc,2.1}^{3}+X_{Glyc}^{3}}\cdot\frac{K_{ATP,2.1}^{4}}{K_{ATP,2.1}^{4}+X_{ATP}^{4}}$ | S5 |
| Maintenance | $v_{2.2}=m_{ATP,2.2}$ | S6 |
| PAA import | $v_{3.1}=k_{perm,3.1}\cdot a_{cell,3.1}\cdot\left(\frac{C_{HPAA}\cdot\rho_{broth}}{1+10^{pH_{ext}-pK}}-\frac{\left(X_{HPAA}/2.5\right)}{1+10^{pH_{int}-pK}}\right)$ | S7 |
| PAA export** | $\frac{dX_{E,3.2}}{dt}=\alpha_{3.2}+\beta_{3.2}\cdot\mu-k_{dE,3.2}\cdot X_{E,3.2}-\mu\cdot X_{E,3.2}$ | S8 |
|  | $v_{3.2}=X_{E,3.2}\cdot X_{PAA}\cdot28.05\cdot10^{-6}$ | S9 |
| Penicillin production | $\frac{dv_{3.3}}{dt}=\frac{\beta_{3.3}\cdot\mu}{1+\left(X_{Glyc}/K_{Glyc,3.3}\right)^{m_{3.3}}}-\left(k_{dE,3.3}+\mu\right)\cdot v_{3.3}$ | S10 |
| Storage/Release Capacity | $\frac{dX_{E,4}}{dt}=\alpha_{4}+\beta_{4}\mu-k_{dE,4}\cdot X_{E,4}-\mu\cdot X_{E,4}$ | S11 |
| Storage | $v_{4.1}=k_{4.1}\cdot X_{E,4}\cdot\frac{C_{S}}{C_{S}+K_{S1,4}}\cdot\left(1+2\cdot\frac{C_{S}}{C_{S}+K_{S2,4}}\right)\cdot\frac{K_{Sto,4.1}}{X_{Sto}+K_{Sto,4.1}}$ | S12 |
| Release | $v_{4.2}=k_{4.2}\cdot X_{E,4}\cdot\frac{K_{S2,4}}{C_{S}+K_{S2,4}}\cdot\left(1+2\cdot\frac{K_{S1,4}}{C_{S}+K_{S1,4}}\right)\cdot\frac{X_{Sto}^{2}}{X_{Sto}^{2}+K_{Sto,4.2}^{2}}\cdot\frac{X_{ATP}^{2}}{X_{ATP}^{2}+K_{ATP,4.2}^{2}}$ | S13 |

* X_i_ are intracellular concentrations in μmol/gDW, C_i_ are extracellular concentrations in mol/kg (broth) and v_i_ are reaction rates in mol/CmolX/h. ** 28.05 gDW/CmolX is the biomass molecular weight and 10^-6^ is the concentration factor from μmol to mol

Table S6. Optimized parameters used in the kinetics of the metabolically structured model (Tang et al. 2017)

| No. | Parameter | Value | St.Err. | Unit |
| --- | --- | --- | --- | --- |
| 1 | q_E,1.1,max_ | 6.50×10^-2^ | 4.41×10^-3^ | U/CmolX/h |
| 2 | δ_0_ | 5.50×10^-2^ | 1.03×10^-3^ | 1/h |
| 3 | k_1.1_ | 0.10 | 1.16×10^-3^ | 1/h |
| 4 | k_dE,1.1_ | 1.46×10^-2^ | 6.55×10^-3^ | 1/h |
| 5 | k_E,1.1_ | 0.26 | 1.54×10^-2^ | molglc/U |
| 6 | K_S,1.1_ | 9.80×10^-6^ | 2.80×10^-6^ | molglc/m^3^ |
| 7 | *v*_1.2,max_ | 0.18 | 7.74×10^-2^ | molC/CmolX/h |
| 8 | K_Glyc,1.2_ | 31.38 | 4.91 | μmolC/gDW |
| 9 | K_AA,1.2_ | 870.23 | 6.96×10^1^ | μmolC/gDW |
| 10 | K_ATP,1.2_ | 2.01 | 2.60×10^-1^ | μmolATP/gDW |
| 11 | *v*_1.3,max_ | 0.32 | 9.92×10^-2^ | molC/CmolX/h |
| 12 | K_Glyc,1.3_ | 38.54 | 9.25 | μmolC/gDW |
| 13 | K_AA,1.3_ | 757.81 | 128.83 | μmolC/gDW |
| 14 | K_ATP,1.3_ | 1.95 | 0.24 | μmolATP/gDW |
| 15 | *v*_2.1,max_ | 0.35 | 9.45×10^-2^ | molC/CmolX/h |
| 16 | K_Glyc,2.1_ | 25.64 | 5.58 | μmolC/gDW |
| 17 | K_ATP,2.1_ | 6.01 | 0.66 | μmolATP/gDW |
| 18 | m_ATP,2.2_ | 3.3×10^-2^ | 1.2×10^-2^ | molATP/CmolX/h |
| 19 | k_perm,3.1_ | 1.62×10^-2^ | 3.74×10^-3^ | m/h |
| 20 | a_cell,3.1_ | 56.00 | - | m^2^/CmolX |
| 21 | α_3.2_ | 0.00 | 0 | 1/h^2^ |
| 22 | β_3.2_ | 1.56×10^3^ | 218.4 | 1/h |
| 23 | k_dE,3.2_ | 0.35 | 4.90×10^-2^ | 1/h |
| 24 | β_3.3_ | 6.50×10^-4^ | - | molPenG/CmolX/h |
| 25 | k_dE,3.3_ | 1.47×10^-2^ | 1.2×10^-3^ | 1/h |
| 26 | K_3.3,Glyc_ | 30.76 | 1.58 | μmolC/gDW |
| 27 | m_3.3_ | 6.00 | - | - |
| 28 | α_4_ | 8.01×10^-4^ | 1.68×10^-4^ | molglc/CmolX/h^2^ |
| 29 | β_4_ | 2.89×10^-1^ | 1.49×10^-2^ | molglc/CmolX/h |
| 30 | k_dE,4_ | 0.29 | 1.23×10^-2^ | 1/h |
| 31 | k_4.1_ | 1.01 | 0.10 | molC/molglc |
| 32 | k_4.2_ | 3.99 | 0.48 | molC/molglc |
| 33 | K_S1,4_ | 1.00×10^-8^ | 1.33×10^-8^ | molglc/m^3^ |
| 34 | K_4,S2,4_ | 1.00×10^-4^ | 1.37×10^-4^ | molglc/m^3^ |
| 35 | K_ATP,4.2_ | 6.48 | 0.9 | μmolATP/gDW |
| 36 | K_Sto,4.1_ | 4.25×10^3^ | 9.32×10^2^ | μmolC/gDW |
| 37 | K_Sto,4.2_ | 7.99×10^3^ | 1.07×10^3^ | μmolC/gDW |
| 38 | pH_ext_ | 6.50 | - | - |
| 39 | pH_int_ | 7.20 | - | - |
| 40 | pK_PAA_ | 4.31 | - | - |
